# Validity Limits of High-Speed Laser-Speckle Modal Measurement on a Rotating Shaft: A Pre-Impact Falsification Test

**DOI:** 10.3390/s26217001

**Published:** 2026-11-05

**Authors:** Jongyun Kim, Seongkeol Kim

**Affiliations:** 1 Department of Mechanical Design and Robot Engineering, Seoul National University of Science and Technology, Seoul 01811, Republic of Korea; 24520007@seoultech.ac.kr; 2 School of Mechanical Systems Engineering, Seoul National University of Science and Technology, Seoul 01811, Republic of Korea

**Keywords:** experimental modal analysis, rotating shaft, high-speed camera, laser speckle, speckle decorrelation, non-contact vibration measurement

## Abstract

**Highlights:**

**What are the main findings?**

- Stationary laser-speckle measurements agreed with contact-reference frequencies within 1.06–2.60%. During rotation, optical candidates were detected in six measurement conditions. In three of these conditions, coherence at the selected candidate frequency was higher before impact than after impact. High coherence alone therefore did not establish a valid modal identification.
- In the tested optical configuration, rotation increased the optical noise floor by 35–46 dB and reduced frame-to-frame speckle correlation from 0.999 to 0.708. The rotating condition with the highest coherence yielded no candidate satisfying the identification criterion within the decision band.

**What are the implications of the main findings?**

- A matched pre-impact control can reveal when apparently high coherence provides insufficient evidence of an impact-induced modal response. The comparison uses the same records and requires no additional sensor when a suitable pre-impact segment is available.
- Passing the control does not establish that an optical candidate is a structural mode. Phase-trend sensitivity and differences between tracking methods require further scrutiny, and the findings remain specific to the shaft, optical configuration and rotational speeds tested.

**Abstract:**

Laser-speckle tracking enables non-contact vibration measurement, but modal identification during rotation remains challenging. This study examines whether resonance-like frequency response functions and high coherence provide sufficient evidence of modal identification. A steel shaft was tested at 0, 100, and 200 rpm using impact excitation at four positions. The assessment combined stationary cross-validation against an accelerometer, matched pre-impact comparisons, and measurement of optical degradation. At 0 rpm, optical frequencies differed from contact-reference values by 1.06% at P2 and 2.60% at P3. The two rotating speeds and four excitation positions produced eight measurement conditions. Two yielded no candidate within the decision band. Six conditions yielded optical candidates. In three of these conditions, coherence at the selected frequency was higher before impact than after impact. Removing a linear phase trend eliminated one additional candidate; however, this outcome varied in leave-one-out tests. Two tracking methods produced candidates 12.21 Hz apart at 200 rpm, but the cause remained unresolved. Rotation increased the optical noise floor by 35–46 dB and reduced speckle correlation from 0.999 to 0.708. Under these conditions, high coherence alone did not validate modal identification. Matched pre-impact comparisons provide an additional check, but passing this check does not confirm a structural mode.

## 1. Introduction

Modal testing of rotating machinery is often performed under stationary conditions. This allows controlled excitation and direct attachment of contact sensors to the shaft. Modal properties may change during rotation because gyroscopic effects and bearing or support conditions can shift or split resonances [1]. Damping may also change [2,3]. Measurements during rotation are therefore needed, but rotational harmonics and sampling artifacts complicate identification [4,5]. Shaft-mounted contact sensors require slip rings or telemetry, and their mass may affect a lightweight rotor.

Optical methods allow vibration measurement without attaching a sensor to the rotating surface. High-speed cameras can measure motion using digital image correlation, phase-based methods, or laser-speckle tracking [6,7]. Recent developments include marker-based measurement systems and benchmark datasets for video-based modal analysis [8,9]. Studies of laser-speckle tracking have examined its accuracy and limitations using projected patterns and directly illuminated surfaces [10,11]. Speckle-related errors have also been investigated in laser vibrometry [12,13], including measurements from moving platforms [14]. These studies show the importance of understanding how the optical configuration affects measurement quality.

Optical measurements have been compared with accelerometers and laser vibrometers in modal tests [15,16,17], and combined contact and non-contact measurement systems have been demonstrated [18]. For rotating shafts, digital image correlation has been validated against laser Doppler vibrometry at speeds up to 6000 rpm [19]. Other applications include torsional vibration measurement [20], deflection-shape measurement on rotating disks [21], comparisons of vibration measurement techniques on rotating fans [22], and fiber-optic sensing of rotating machinery [23]. These results demonstrate the potential of optical methods. They also motivate a practical question: what evidence is needed to accept a modal identification when an independent reference is unavailable during rotation?

A peak in a frequency response function (FRF) and a high coherence value may appear to provide that evidence. However, coherence describes the linear relationship between the measured input and output. It does not establish that an apparent resonance corresponds to a structural mode. In the present measurements, high coherence also occurs when the optical response window is taken before the impact. This window contains rotation-related motion but no response caused by the impact under analysis. Comparing it with the impact-response window therefore provides a direct check on the interpretation of coherence.

Previous optical studies have assessed agreement with reference measurements and consistency among identified modes. Kalybek et al. compared optical modal parameters with accelerometer measurements [15]. Other studies used contact or optical reference systems [16,17,19]. Li et al. used spatial sampling consistency to identify physical modes [24]. Azizi et al. improved subpixel motion estimation [25], and Deng et al. combined optical measurements, accelerometer data and automated clustering [26].

Data-driven methods have also been applied to pipeline leak detection using induced-leak enhanced scalograms and deep learning [27], and to mechanical-property prediction of fused deposition modeling (FDM)-printed acrylonitrile styrene acrylate (ASA) using experimentally validated ensemble learning [28]. These applications provide broader context for data-driven engineering assessment, while the present study examines the measurement evidence supporting optical modal identification during rotation.

The question addressed here is how far optical FRFs and coherence measured on a rotating shaft can be accepted as evidence for modal identification. Reference agreement and consistency among identified modes address important aspects of validation [15,16,17,19,24,25,26]. A complementary check is whether coherence supporting a candidate also occurs before the applied impact. We compare matched impact-response and pre-impact windows from the same recording. The pre-impact segment contains rotation-related fluctuations but no response caused by the impact under analysis. This comparison can reveal insufficient evidence for an impact-related modal interpretation. Passing it does not establish a structural mode.

This study makes three contributions. First, optical and contact measurements are compared under stationary conditions using the same physical impacts. Second, a matched pre-impact control is applied during rotation using the same impacts, window length, and processing procedure as the impact-response analysis. Third, changes in the optical noise floor and frame-to-frame speckle correlation are measured to assess the degradation of the optical measurement. These contributions are distinct from the three assessment steps applied to each rotating condition in Section 3.3.

The experiments use a steel shaft tested at 0, 100, and 200 rpm, with impact excitation at four positions. Laser-speckle motion is recorded from the shaft end face. All four excitation positions are included in the analysis, while the stationary frequency comparison is made at P2 and P3, where both measurement channels satisfy the identification criterion. The study focuses on the transverse resonance near 235 Hz, referred to as the first bending mode. Its mode shape is not measured. The experiment provides a case in which high coherence does not establish valid optical modal identification. It does not determine a general rotational-speed limit or validate the pre-impact test across machines. A matched pre-impact control helps reveal this limitation, while further validation is needed for candidates that remain.

## 2. Materials and Methods

### 2.1. Test Rig and Excitation Positions

**Figure 1:**
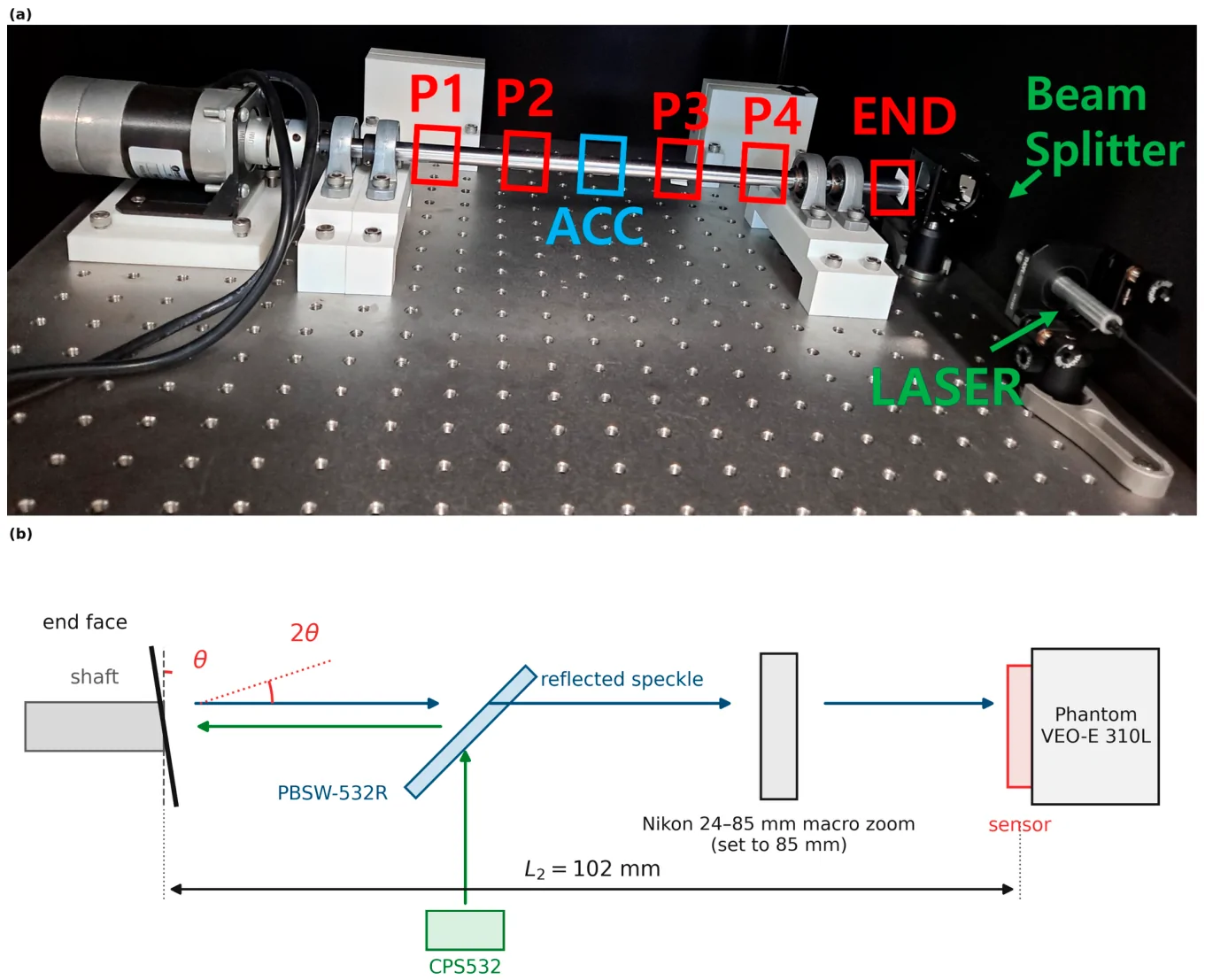
Experimental arrangement. (**a**) Test rig and excitation positions: a 400 mm × 12 mm S45C-grade carbon steel shaft driven from the left and supported at two bearing stations, each of two adjacent pillow-block units, with the impact positions P1 at x = 320 mm, P2 at 260 mm, P3 at 160 mm and P4 at 100 mm marked in red, the accelerometer position ACC in blue, and the motor-opposite end face marked END, from which x is measured. The accelerometer is on the shaft at x = 210 mm at 0 rpm and on the bearing housing under rotation, as Section 2.2 explains. (**b**) Optical path. L_2_ is the nominal free-space optical lever from the end face, used only to define the geometric-equivalent angular scale of Section 2.2.

The shaft is a 400 mm long, 12 mm diameter S45C-grade carbon steel shaft supported at two bearing stations centered at x = 50 mm and x = 350 mm, each station comprising two adjacent bearing units on a common base, giving four rolling-element bearings in total. The axial coordinate x is measured from the motor-opposite end face, and the shaft is driven from the far end. Four impact positions were used, at x = 320 mm (P1), 260 mm (P2), 160 mm (P3) and 100 mm (P4). P1 and P4 are approximately symmetric about the bearing-span center, as are P2 and P3. The shaft was tested at 0 rpm, 100 rpm, and 200 rpm. Figure 1 shows the experimental arrangement and optical path.

### 2.2. Instrumentation

At 0 rpm, the accelerometer was mounted on the shaft at x = 210 mm and recorded the same impacts as the optical system. These records support stationary optical–contact frequency comparison. During rotation, the accelerometer was mounted on the bearing housing. A separate stationary housing measurement provides the baseline for comparing housing responses across speeds. Because the housing acceleration and optical shaft response represent different responses at different locations, the housing measurements do not directly validate rotating optical candidates. Those candidates are assessed using the matched pre-impact comparison and phase-trend test.

Excitation was applied with a Dytran 5850B impulse hammer (Dytran Instruments, Chatsworth, CA, USA) with a steel tip and a sensitivity of 22.48 mV/N. A PCB Piezotronics 352C33 accelerometer (PCB Piezotronics, Depew, NY, USA) measured the contact response. Force and acceleration were acquired using an IOtech 650u data-acquisition (DAQ) unit (IOtech, Cleveland, OH, USA) operated with eZ-Analyst software (version 5.1.140) at 5120 Hz in blocks of 8192 samples, giving 0.625 Hz resolution. The trigger was at sample 836. Optical analysis uses 4096 samples at 5000 frames per second at 0 rpm and 8192 samples at 20,000 frames per second during rotation, giving resolutions of 1.221 and 2.441 Hz. Independently triggered stationary contact and optical records were paired by acquisition order within each run. Counts matched at each position. Instrument specifications and acquisition checks are provided in Supplementary Section S12.

A Thorlabs CPS532 laser (Thorlabs, Newton, NJ, USA) illuminates the motor-opposite shaft end face at 532 nm through a polarizing plate beamsplitter. Illumination and imaging axes are collinear. The face carries retroreflective tape, and a 532 nm bandpass filter is fitted to the lens. A Phantom VEO-E 310L camera (Vision Research, Wayne, NJ, USA) records the backscattered speckle pattern at 256 × 256 pixels with a 30 µs exposure. The Nikon 24–85 mm macro zoom (Nikon Corporation, Tokyo, Japan) is set to 85 mm and f/22. The optical path from the end face is approximately 9.5–10 cm. The geometry and exposure were fixed across conditions. Figure 1 shows the optical path. Figure S9 and Supplementary Section S12 report the recorded pattern, speckle size, clipping and acquisition settings.

The optical channel does not measure translation of the end face. A tilt of the face by an angle θ deflects the reflected beam by 2θ, and the pattern translates accordingly at the sensor. Using the free-space lever relation as a geometric equivalent, the nominal angular scale is *s_θ_* = p/(2L_2_) = 49.02 µrad per pixel, with pixel pitch p = 10 µm and nominal lever length L_2_ = 102 mm. The optical reference planes of this defocused imaging path were not established, so L_2_ is not a calibrated sensor-path length, and the optical signal is reported in pixels throughout. The equivalent angular trajectory and the equivalent angular frequency response function are given by Equations (1) and (2) respectively, in which *y*_px_(*t*) is the tracked optical trajectory in pixels and *H*_px*F*_(*f*) is the measured pixel-per-force frequency response function:

*θ*_eq_(*t*) = *s_θ_y*_px_(*t*) (1)

*H_θF_*_,eq_(*f*) = *s_θ_H*_px*F*_(*f*) (2)

The equivalent spectral-density conversion is *S_θ_*_,eq_(*f*) = *s*^2^*_θ_ S*_px_(*f*). All primary results remain in pixels or pixels squared per hertz because no independent angular calibration was performed. Frequency, damping ratio and coherence are invariant to a constant scale factor. Supplementary Section S12 gives the optical-path assumptions.

### 2.3. Speckle Tracking

**Figure 2:**
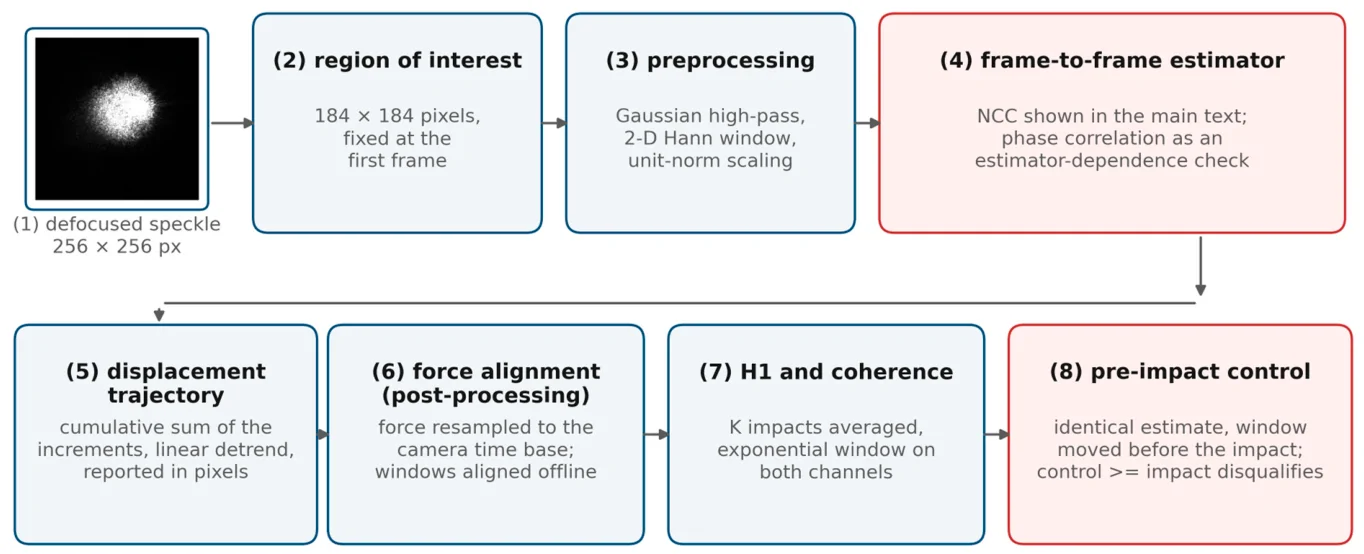
Optical processing chain. A defocused speckle pattern from the end face is tracked frame to frame inside a fixed region of interest. The increments are accumulated into a displacement trajectory in pixels, aligned in post-processing with the measured hammer force, and reduced to an H1 estimate and its coherence. The final step recomputes the identical estimate on a window taken entirely before the impact, which is the falsification test of Section 2.7. Normalized cross-correlation is the estimator shown in the main text. The two phase-correlation variants are applied to the same recordings as a check on estimator dependence.

Frame-to-frame motion was estimated within a 184 × 184 pixel region of interest (ROI), fixed at its initial position on the sensor. The ROI does not follow the moving surface. Figure S9 shows its placement within the recorded image. Figure 2 summarizes the processing sequence.

The same preprocessing is applied before each tracker: subtract a Gaussian-blurred image with a standard deviation of four pixels, apply a two-dimensional Hann window, and normalize to unit Euclidean norm. No clipping mask is applied. The speckle size and saturated pixels are characterized in Supplementary Section S12.

Normalized cross-correlation (NCC) provides the main results [29,30]. Its stationary estimates were compared with the contact measurements from the same impacts at P2 and P3. Phase correlation (PHCORR) normalizes the cross-power spectrum by its magnitude before inversion [31]. Phase correlation with subpixel refinement (SKPCC) uses the phase-correlation framework with subpixel refinement [32]. These two variants assess tracker dependence. Their candidate results and included impact counts are reported in Table S1.

The trackers return frame-to-frame increments. The increments are cumulatively summed to obtain a trajectory, and a first-order polynomial is removed to suppress accumulated linear drift. The resulting time signal is then processed as described in Section 2.4.

### 2.4. Signal Processing

Force and acceleration contain a mains comb at multiples of 60 Hz. It is removed by least-squares subtraction of 42 harmonics with quadratically time-varying amplitudes, fitted only to samples without an impact response. Tests with synthetic responses showed frequency changes of at most one spectral line and decreases of 1.5–3.3 percentage points in the half-power damping ratio. The damping estimates therefore carry a systematic processing uncertainty and are not used to validate agreement between channels. The optical channel has no corresponding mains-comb subtraction. Supplementary Section S13 reports the tests.

The force record, sampled at 5120 Hz, is resampled onto the camera time base by anti-aliased polyphase resampling with the exact integer ratio. Force and acceleration were acquired by the same DAQ and triggered by the hammer force signal, while the camera was triggered independently, so their records were aligned in post-processing [16,33]. At 0 rpm, the analysis window in each channel starts 40 samples before an onset located in that same channel, with no further fractional-delay optimization. Under rotation, the residual inter-channel delay was refined for each shot over 200–300 Hz by searching ±12 samples in 0.05-sample increments, referred to the force. Because that interval contains the decision band, the identical delay-estimation procedure is run independently on the pre-impact control window so that the two windows receive matched processing, and any gain in in-band phase consistency introduced by the alignment is available to both. Supplementary Section S13 defines the onset detectors and reports the cost of mispositioning the window. Table S8 reports sensitivity to the residual-delay fitting band (Supplementary Section S11).

Each measurement condition is defined by one combination of shaft speed and excitation position within the channel or image-registration tracker under discussion. The FRF is estimated with the H1 estimator over the K impacts available in that measurement condition. Shot-to-shot time alignment is calculated relative to the force rather than to an arbitrarily chosen reference shot, which keeps the phase consistent in the averaged estimate. Because no common electrical trigger existed, the absolute phase of the optical estimate is not established here. The contact channel is measured as acceleration, so its H1 estimate is divided by (2πf)2 and scaled to millimeters per newton, while the optical estimate remains in pixels per newton. With *X_k_*(*f*) and *Y_k_*(*f*) the force and response spectra of the k-th impact and $X_{k}^{*}$(*f*) the complex conjugate of *X_k_*(*f*), the estimator and the ordinary coherence are given by Equations (3) and (4) respectively:

(3) $$H_{1}\left(f\right)\;=\;\frac{\sum_{k}Y_{k}\left(f\right)X_{k}^{*}\left(f\right)}{\sum_{k}{\left|X_{k}\left(f\right)\right|}^{2}}$$

(4) $$\gamma^{2}\left(f\right)=\frac{{\left|\sum_{k}Y_{k}\left(f\right)X_{k}^{*}\left(f\right)\right|}^{2}}{\sum_{k}{\left|X_{k}\left(f\right)\right|}^{2}\sum_{k}{\left|Y_{k}\left(f\right)\right|}^{2}}$$

No band-pass, low-pass, high-pass or notch filter is applied to the time signals used for FRF estimation. The onset detector uses a band-pass signal only to locate the response onset. The anti-aliasing operations precede sampling or resampling [34]. Image preprocessing is described separately in Section 2.3. Filtering only the response could change the FRF, and non-causal filtering could spread the impact response into the pre-impact segment.

An exponential window e − t/τ is applied to force and response [35,36], with τ chosen so that it has decayed to 1% at the end of the record, giving 88.9 ms for the rotating records and 177.9 ms at 0 rpm. Its apparent damping contribution is given by Equation (5), in which *f*_r_ is the identified resonant frequency:

(5) $$\zeta_{\mathrm{add}}\;=\;\frac{1}{2\pi f_{r}\tau }$$

The window adds 0.758 and 0.379 percentage points to the damping ratios for the rotating and stationary records, respectively. These contributions are subtracted from the reported damping ratios. The resonant frequency is unaffected. A force window was evaluated but is not used, as reported in Supplementary Section S13.

### 2.5. Modal Parameter Extraction

Candidate detection combines FRF phase and magnitude. A local maximum of the signed windowed phase decrease *R*(*f*) must reach 45°, and a local FRF magnitude maximum must lie within four spectral lines of that phase-change maximum. The candidate frequency f* is the frequency of the magnitude maximum, not necessarily the phase-change maximum. Four lines correspond to 2.5 Hz for stationary contact measurements and 9.8 Hz for rotating optical measurements. The rotating values are therefore treated as candidates, not fully resolved resonances. Equation (6) defines the phase-change quantity [37].

*R*(*f*) = *φ*(*f* − *h*) − *φ*(*f* + *h*) (6)

Here *φ*(*f*) is the unwrapped FRF phase in degrees, and *h* is the positive phase-window half-width. The reported half-width is 10 Hz. In the discrete calculation, *h* is rounded to the nearest frequency bin. For the rotating records, four bins give an effective half-width of 9.766 Hz. A constant phase offset cancels, but a linear phase trend contributes the amount in Equation (7).

*φ_lin_*(*f*) = *af* + *b*, *R_lin_*(*f*) = −2*ha* (7)

The slope *a* is in degrees per hertz, and *b* is a phase offset in degrees. For the discrete calculation, *h* in Equation (7) is the effective bin-rounded half-width. The contribution is constant across frequency and has the opposite sign to *a*. Equation (8) applies at the phase-change maximum associated with the candidate.

*R*(*f*) ≥ 45° (8)

The threshold is not applied to the absolute value of *R*(*f*). A smooth phase trend can contribute enough phase change to satisfy Equation (8), even without a localized resonance feature. The phase requirement alone therefore does not establish a structural mode. Section 3.3 tests this dependence by subtracting a fitted linear trend before recalculating *R*(*f*). The corresponding before-and-after comparison is shown in Supplementary Figure S10.

For the stationary P2 and P3 comparisons, varying the phase-window half-width from 6 to 20 Hz left the frequency unchanged whenever a candidate was detected. Some contact candidates were absent at two settings. Simple magnitude peak picking returned the same four stationary frequencies. These checks and the phase curves are provided in Supplementary Section S9 and Figure S7. Rotating sensitivity results are summarized in Section 3.3.

The damping ratio is estimated from the half-power bandwidth of the averaged FRF [37], with the exponential-window contribution removed. The spectral resolution is 1.221 Hz for stationary optical data, 0.625 Hz for contact data and 2.441 Hz for rotating optical data. This resolution limits separation of nearby features. It is not a statistical confidence interval.

A single-degree-of-freedom circle fit was also evaluated but not used. Over the wider interval needed for that fit, the neighboring pole drew the estimate towards approximately 228 Hz optically and 239–241 Hz on the contact channel. Reported frequencies therefore use the phase and local-maximum criterion, and damping uses the half-power bandwidth.

### 2.6. Optical Noise Floor and Speckle Correlation

The optical noise floor and frame-to-frame speckle correlation are evaluated separately. For the noise-floor estimate, a late stationary segment or a pre-impact rotating segment is selected without the target impact response. After removing the segment mean, the single-sided power spectral density (PSD) is calculated using Equation (9). *Y*(*f*) is the unwindowed discrete Fourier transform, *N* is the segment length, and *f*_s_ is the sampling frequency.

(9) $$S\left(f\right)=\frac{2{\left|Y\left(f\right)\right|}^{2}}{Nf_{s}}$$

PSD is reported in pixels squared per hertz. The band level is the mean density over its frequency lines, and the condition-level value is the median across impacts. Stationary and rotating segments have equal duration (0.4096 s) and frequency resolution (2.441 Hz). This normalization allows comparison across frame rates but does not eliminate frame-rate effects on tracking.

Two bands carry measurements. The first is the decision band, 225–247 Hz, defined in Section 2.7. The second is 400–700 Hz, a comparison band in which the contact channel shows no prominent resonance: the accelerometer receptance at 0 rpm contains no local maximum of prominence 3 dB or more between 300 and 708 Hz at any position. Comparing the two isolates, a broadband change from one is confined to the modal band.

The second quantity is the frame-to-frame speckle correlation, the maximum of the cross-correlation surface between consecutive frames, each ROI being preprocessed exactly as in Section 2.3. The value quoted for a record is the median over its frames, and that for a condition is the median over its records.

### 2.7. Validity Criteria and the Pre-Impact Control Test

The reported decision band is 225–247 Hz. It is centered on the stationary first-mode estimate near 236 Hz and ends below the neighboring pole at 248.0–248.5 Hz. Its placement uses the stationary’s results, not the rotating’s outcomes. Candidate maxima are searched over 200–300 Hz and then restricted to the decision band. Figures generally display 150–400 Hz. Section 3.3 deliberately varies the band for sensitivity analysis. Other reported candidate results use 225–247 Hz.

Coherence is reported both at its maximum within the decision band and at the selected candidate frequency. These are different comparisons. For N averaged records, approximately 1/N is shown as the null expectation of ordinary coherence for unrelated signals. It is a reference level, not a significance threshold, and is not used for candidate acceptance. Magnitude maxima must have a prominence of at least 0.5 dB to avoid treating a band-edge maximum of a monotonic spectrum as a resonance.

The pre-impact comparison uses the same impacts, window length, force records and processing procedure as the impact-response estimate. Only the response window is moved entirely before the impact. If coherence at f* does not exceed the pre-impact value, that coherence provides insufficient evidence for an impact-excited modal interpretation. If it exceeds the pre-impact value, the candidate is not disqualified by this check but remains unconfirmed. This diagnostic is demonstrated for the tested configuration only. A separate comparison of the two band maxima assesses the band-search procedure rather than a common frequency. Results for both comparisons are provided in Table S9.

The frequency-domain figures of Section 3 are shown over 150–400 Hz, where the first mode lies. Full-band stationary and rotating optical results are shown in Figures S1 and S2, respectively, and the measured hammer-force spectrum is shown in Figure S5c, so that a reader can judge whether other modes are present and where the noise floor sits.

Impact quality was evaluated from the force record using the secondary-force ratio: the largest force within 10 ms after the first pulse decayed below 2% of its peak, divided by that peak. Shots at 0.20 or above were flagged for the sensitivity analysis of Section 3.6. Results are never pooled across excitation positions. Two considerations of different kinds determine which stationary comparisons can be formed. The first is independent of any modal result: only the second of the two acquisition runs has the illuminated spot at the same sensor position at every excitation point. The second is an outcome of the criterion: both channels return a candidate only at P2 and P3, since at P1 the contact channel returns none while the optical channel returns 236.816 Hz, and at P4 the reverse holds. All four positions form the analysis population and are reported in Table S4 (Supplementary Section S8).

Generative AI tools were used during manuscript preparation to assist with language editing and consistency checking.

## 3. Results

### 3.1. Stationary Validation Against the Contact Reference

**Figure 3:**
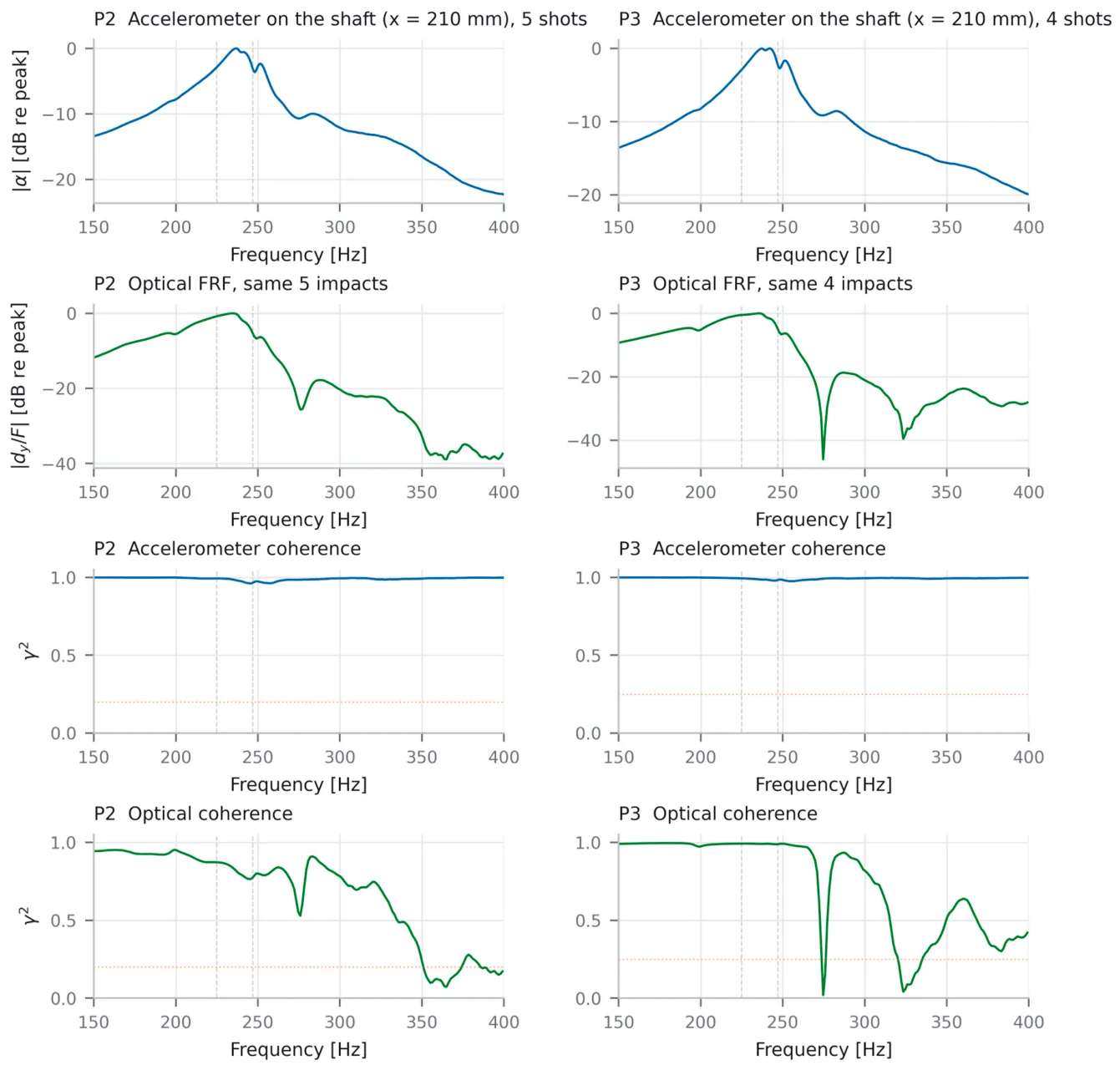
At 0 rpm, P2 (**left**) and P3 (**right**) over 150–400 Hz, from the same physical impacts recorded by the two independently triggered systems. Rows from top: receptance of the accelerometer on the shaft at x = 210 mm, optical FRF, accelerometer coherence, optical coherence. Dashed vertical lines bound the decision band. The dotted horizontal line on each coherence panel is the approximate null expectation 1/N.

Figure 3 compares the two channels at 0 rpm at P2 and P3, from the same physical impacts recorded by the two independently triggered systems. In each of the four measurement conditions, the phase of the averaged FRF sweeps through the band and a magnitude maximum coincides with it, so the mode is identified by the criterion of Section 2.5. The contact channel places it at 236.875 Hz at P2 and 241.875 Hz at P3, and the optical channel at 234.375 and 235.596 Hz, differences of 1.06% and 2.60% against an optical resolution of 1.221 Hz. At the identified frequency, the coherence is 0.981 and 0.983 on the contact channel and 0.840 and 0.990 optically. The in-band maxima are higher but occur at the lower edge of the band rather than at the mode because at 0 rpm, the coherence is high across the whole interval. The value at the identified frequency is the one reported. Table 1 gives both channels at both positions.

Two aspects require qualification. The damping ratios are tabulated for completeness but are not compared between the channels, because the accelerometer record passes through the mains-comb subtraction in Section 2.4, while the optical record carries no mains content and is not treated. The two channels are therefore compared on frequency alone. There, the contact value at P3, 241.875 Hz, sits close to the neighboring pole and its peak is the broadest of the four positions, so the contact reference is itself less sharply defined at P3 than at P2. The 2.60% difference is as much a property of that reference as of the optical channel, and P2 is the stronger of the two stationary comparisons.

The records used are those of the second of the two acquisition runs collected in the experiment, the only acquisition run in which the illuminated spot occupied the same sensor position at every excitation point. That criterion is the recorded ROI position and is independent of any modal result. Because the averages include impacts exceeding the impact-quality threshold of Section 2.7, the identical estimate was recomputed on the clean impacts alone, and all four identified frequencies lie within one spectral line of the full-average values, so the stationary identification is not an artifact of the repeated impacts. Supplementary Sections S4 and S8 report the impact-quality analysis and the results at all four positions in full. Figure S6 shows the stationary comparison with both acquisition runs pooled.

### 3.2. Contact-Channel Response Under Rotation

Using the housing baseline defined in Section 2.2, the criterion returns candidates at all four positions at 0 rpm, three positions at 100 rpm and one position at 200 rpm (Table 2). These results describe the housing response and are not a direct reference for the rotating optical shaft response.

Relative to the stationary housing baseline at each position, the apparent damping increases by 3.6–10.3 percentage points. Candidate frequencies decrease by up to 9.4 Hz except at 100 rpm P2. The damping changes are descriptive because mains-comb subtraction can bias the half-power estimate. No position-to-position modal consistency or shaft-response validation is inferred from these housing data.

### 3.3. Optical Identification Under Rotation

With NCC, the criterion returns no candidate in two of the eight rotating conditions, one candidate in five, and three candidates at 100 rpm P1. Candidate frequencies span 227.05–244.14 Hz. For context, housing candidates span 228.75–235.63 Hz at 100 rpm, with only one candidate, 226.88 Hz at P3, at 200 rpm. These are responses at different locations. In six rotating optical conditions, the maximum FRF magnitude within the band occurs at its lower edge, which is insufficient evidence of a resonance.

Phase correlation [31,38], analyzed with the same criterion and windows, returns candidates in seven of the eight measurement conditions, with frequencies from 227.05 to 246.58 Hz. The subpixel variant returns a candidate in two of the four conditions available to it. The impact sets differ between tracking methods because an impact is included only when both analysis windows fall within the recorded displacement data. Table S1 lists the impact counts and candidates for each method. For example, at 100 rpm P2, normalized cross-correlation and the subpixel variant yield no candidates, whereas phase correlation yields candidates at 227.05 and 236.82 Hz. Figure S2 shows the phase-correlation result over the full band.

**Figure 4:**
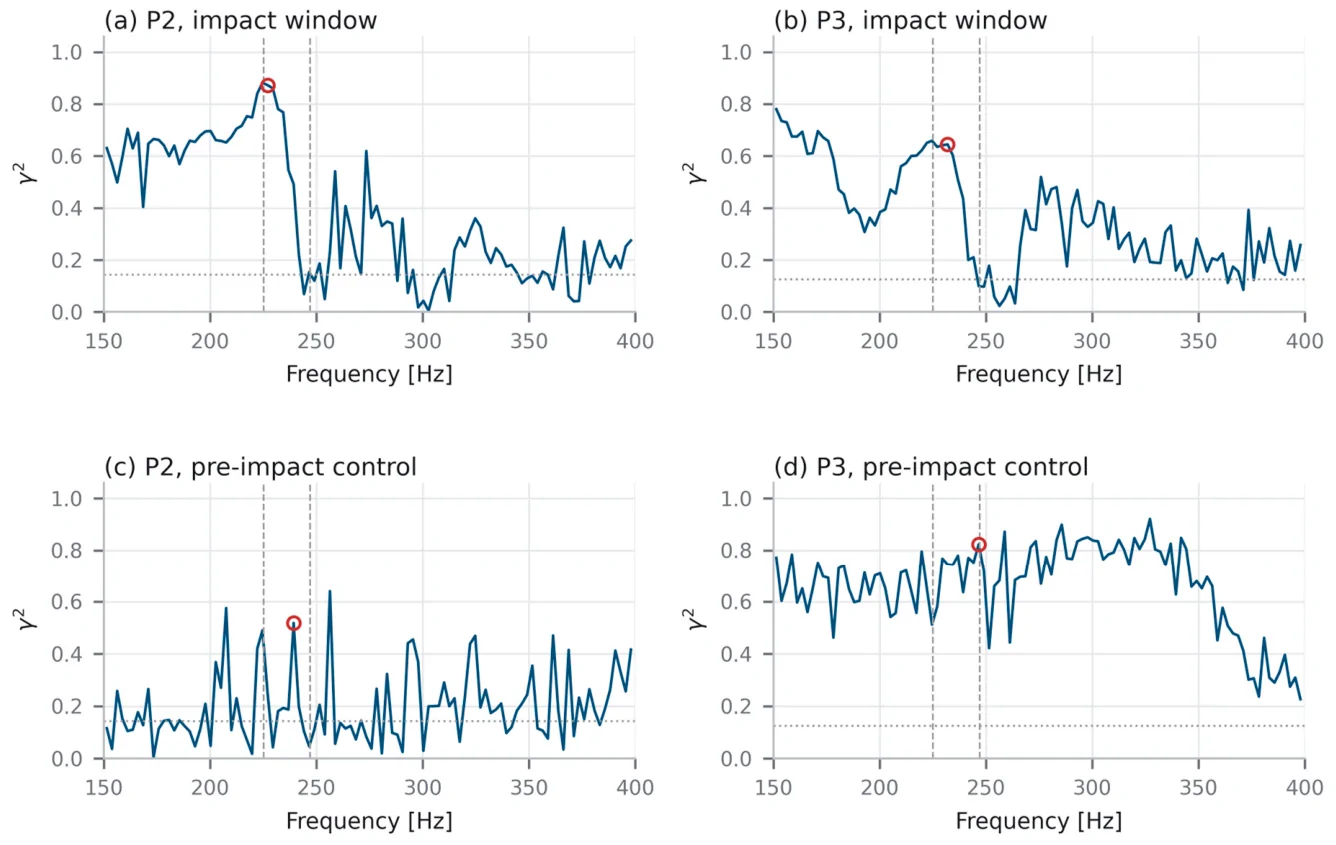
The pre-impact control test at 100 rpm with the normalized cross-correlation tracker. (**a**,**b**) Coherence is calculated from the impact-response window. (**c**,**d**) The same estimate is calculated after moving the response window entirely before the impact. Both calculations use the same impacts and window length. Dashed vertical lines bound the decision band, and the dotted horizontal line is the approximate null expectation 1/N. Circles mark the in-band maximum: 0.872 against 0.518 at P2, seven shots, and 0.645 against 0.824 at P3, eight shots, so P3 is disqualified on the band comparison.

Figure 4 illustrates the pre-impact comparison at 100 rpm. Comparing band maxima disqualifies four of eight NCC conditions, whereas comparing both windows at the selected candidate frequency disqualifies three of the six conditions with a candidate. Table S9 distinguishes these comparisons. Table S1 gives the corresponding tracker-specific results. No pre-impact comparison was performed for SKPCC.

At the selected candidate frequency, pre-impact coherence exceeds impact-response coherence at 100 rpm P1, 100 rpm P3 and 200 rpm P2. The remaining NCC candidates are 227.05 Hz at 100 rpm P4, 236.82 Hz at 200 rpm P3 and 229.49 Hz at 200 rpm P4. They require further assessment rather than being accepted as structural modes.

Table 3 summarizes candidate detection, the pre-impact comparison at the candidate frequency, and the phase-trend test for each rotating measurement condition. These assessment steps are distinct from the three contributions stated in Section 1. Failure of an assessment indicates insufficient evidence for modal identification, not the absence of a structural mode.

Sensitivity was evaluated for all 36 combinations of three phase thresholds (30°, 45°, 60°), three window half-widths (8, 10, 12 Hz) and four decision bands (225–247, 220–252, 230–242, 215–257 Hz) across eight rotating conditions. In comparisons changing one setting at a time, only the 200 rpm P3 candidate was lost when the threshold rose to 60°. Window-width changes affected 100 rpm P1, 100 rpm P3 and 200 rpm P3. Candidate frequencies did not move when the candidates remained detectable. Narrowing the band excluded frequencies outside it, including both P4 candidates. Widening it admitted additional candidates, including 251.46 Hz at 200 rpm P4 above the neighboring pole. Exclusion by a band boundary is not evidence against a candidate, and additional candidates are not established speed-dependent shifts. Supplementary Section S9 describes the supplied numerical results. These analyses do not establish greater stability of retained candidates. Selected one-setting-at-a-time comparisons are illustrated in Supplementary Figure S11.

To assess phase-trend dependence, a straight line was fitted to the unwrapped phase and removed before repeating the phase criterion and magnitude-coincidence rule. Fitting interval A uses 150–200 and 300–400 Hz. Interval B uses 120–200 and 300–450 Hz. Both exclude the 200–300 Hz search neighborhood to reduce contamination by the resonance region. They are sensitivity checks, not independent confirmation of a mode. Table S5 reports results for both fits. The change in the accepted phase-change quantity is visualized in Supplementary Figure S10.

For NCC, the five candidates at 100 rpm P1, P3, and P4 and 200 rpm P2 and P4 remain after both fits. At 200 rpm P3, the accepted phase-change maximum falls from 48.2° to 19.5° under fit A and 24.5° under fit B. These values use the same original phase-change peak and window. The full-dataset candidate is therefore not retained. This outcome depends on individual impacts, as Section 3.6 shows. PHCORR at 100 rpm P1 is also not retained under either fit (Table S5).

At 200 rpm P4, NCC returns 229.49 Hz while PHCORR returns 241.70 Hz, a difference of 12.21 Hz. Thus, a candidate that survives the NCC control and phase-trend checks does not yield a consistent frequency across the two tracking analyses. Both averages contain eight impacts, but their individual impact identifiers were not verified as identical. The PHCORR displacement records were unavailable for that check, so the discrepancy cannot be attributed solely to the tracker. At 100 rpm P4, the 4.88 Hz difference is additionally confounded by unequal impact counts (seven for NCC and six for PHCORR). Neither comparison supplies an independent reference for the shaft response.

In the stationary comparisons, both P2 candidates and the P3 contact candidate remain under both phase-trend fits. The P3 optical candidate remains only under fit B. P2 therefore provides the stronger stationary comparison (Table S5).

**Figure 5:**
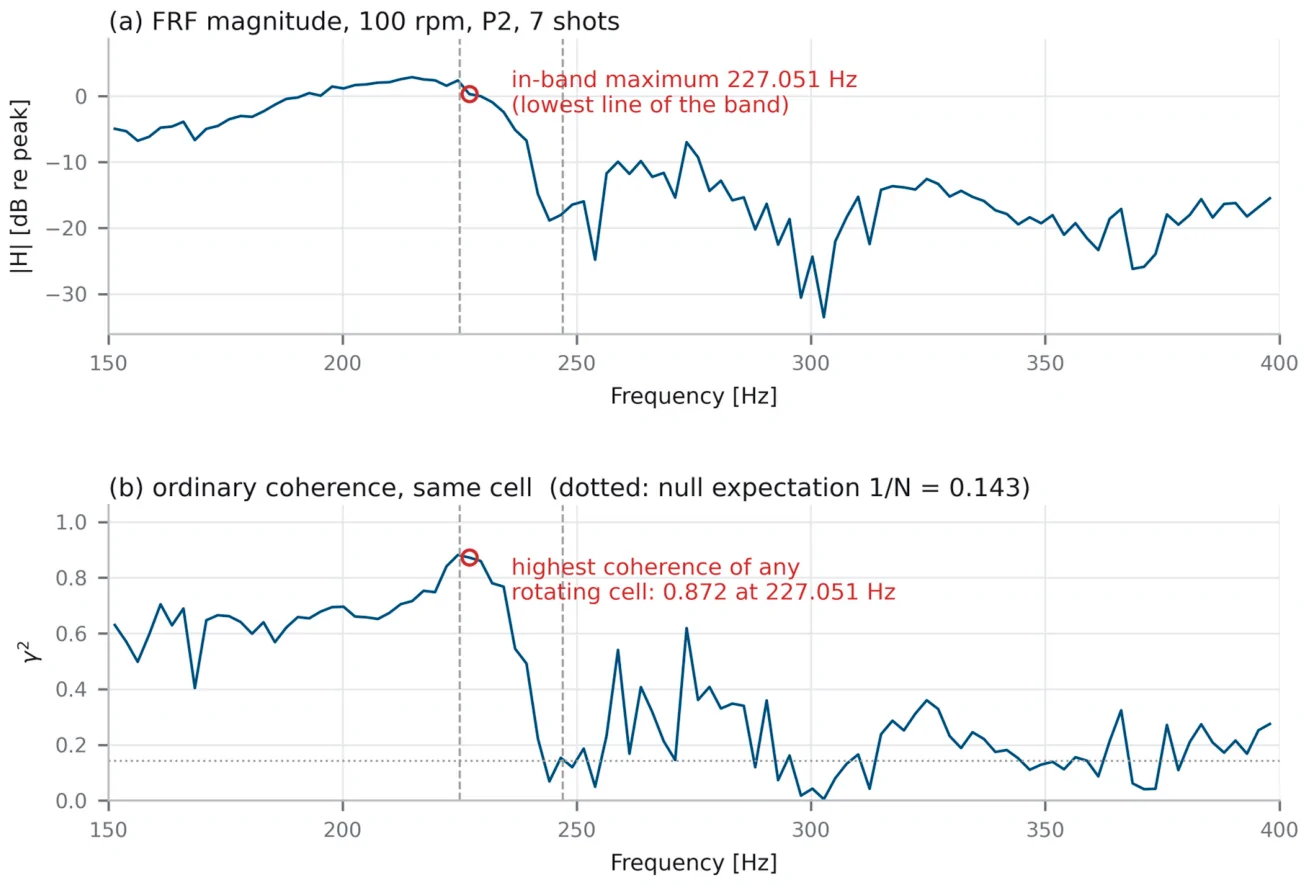
The measurement condition with the highest rotating coherence, 100 rpm at P2, seven shots. (**a**) FRF magnitude: the broad maximum lies near 215 Hz, below the decision band marked by the dashed lines, and the magnitude falls monotonically through the band, so the in-band maximum is pinned to its lowest line. (**b**) Ordinary coherence for the same measurement condition, the highest of any rotating measurement condition, occurring at that same band edge rather than at the frequency of the mode.

The highest rotating band-maximum coherence occurs at 100 rpm P2: 0.872 after impact versus 0.518 before impact. Nevertheless, no candidate is detected within the decision band. Figure 5 shows a broad magnitude maximum near 215 Hz followed by a decrease through the band. This example demonstrates why a high coherence value and a favorable band-maximum comparison do not establish modal identification.

### 3.4. Optical Noise Floor and Speckle Decorrelation

In equal-duration segments without the target impact response, the decision-band optical noise floor rises by 35.4–45.7 dB during rotation. The margin between the impact-response level and the floor decreases from 40.0–45.1 dB at 0 rpm to 6.7–15.2 dB during rotation. Section 2.6 defines the duration and density normalization.

**Figure 6:**
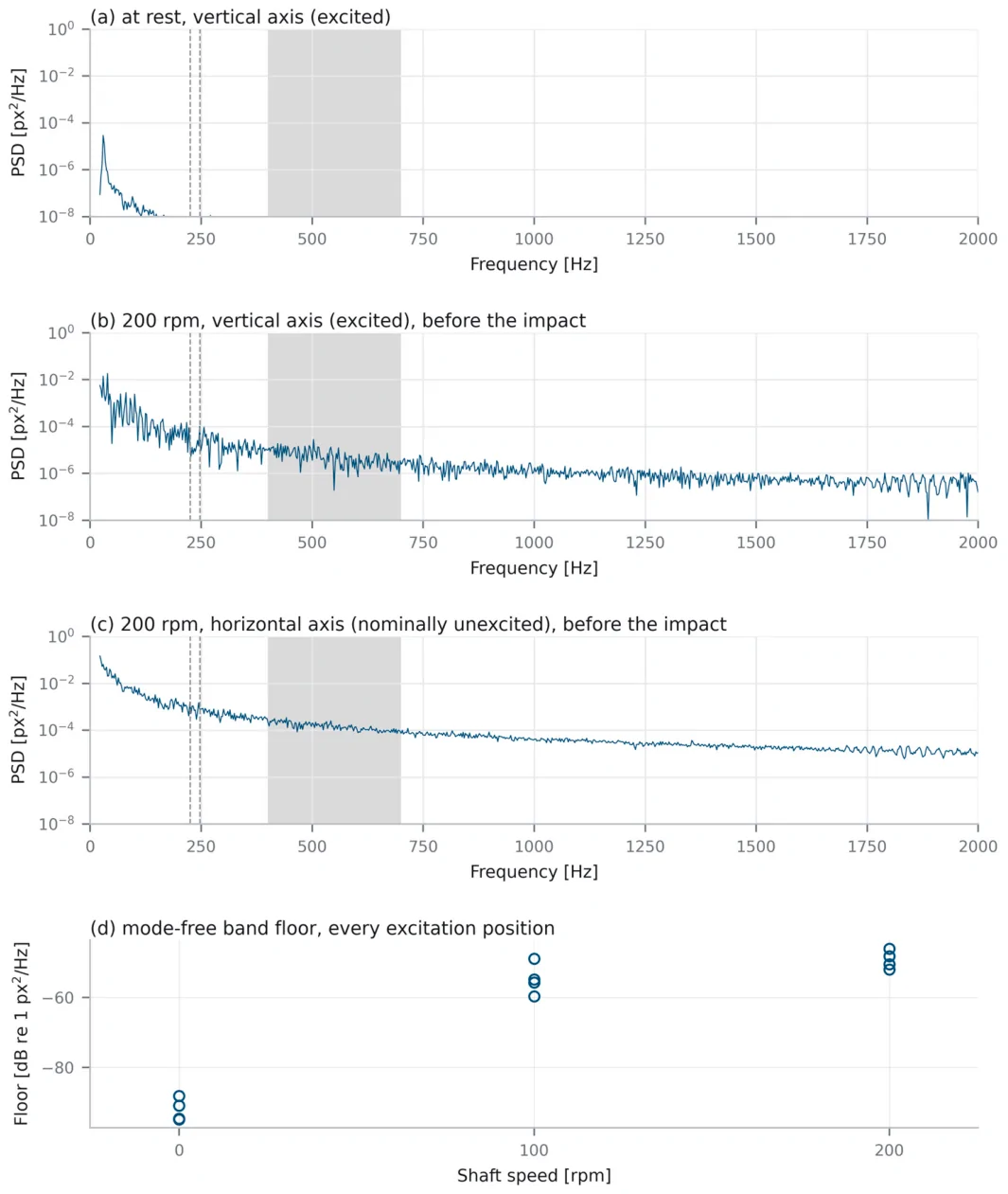
Optical noise floor, measured on windows of 0.4096 s that contain no response. (**a**) At 0 rpm, vertical image axis, taken from the tail of the record. (**b**) At 200 rpm, vertical axis, taken before the impact. (**c**) At 200 rpm, horizontal axis, the nominally unexcited orthogonal image axis. Panels (**a**–**c**) use common axis scales. Dashed vertical lines bound the decision band, and the shaded interval is the comparison band 400–700 Hz, in which the contact channel shows no prominent resonance. (**d**) The comparison-band level at every excitation position and speed.

Three observations support broadband tracking degradation as a major contributor under the tested conditions. First, the 400–700 Hz comparison-band increase is 32.7–46.2 dB, within 2.7 dB of the decision-band increase at every position. Second, the elevated floor also occurs in the nominally unexcited horizontal image axis, where it is 2.8–14.3 dB higher than in the vertically excited axis. Third, frame-to-frame speckle correlation decreases from 0.999 at 0 rpm to 0.822 at 100 rpm and 0.708 at 200 rpm [39]. Figure 6 compares the spectra and band levels. Together, these observations support optical tracking degradation, but do not isolate a single physical cause.

Fixed-ROI overlap loss was much smaller than the measured correlation decrease. Grouping the rotating increments in fours changed the comparison-band level by only 0.0–0.2 dB. These processing checks are detailed in Supplementary Section S13. Grouping does not reproduce recording at a different frame rate or exposure. Because frame rate differed between stationary and rotating acquisitions, the observed degradation cannot be attributed exclusively to rotation.

Rotation orders are also present but cannot be separated reliably within the short transient-response window [40]. Their presence alone does not explain the broadband evidence above. Supplementary Section S5 reports the order-resolution and synchronous-subtraction analyses.

### 3.5. Order Filtering

A multi-order Vold–Kalman filter [41,42,43,44] was tested by adding a synthetic decaying 236 Hz response to a measured pre-impact segment. The most favorable tested configuration retained only 18.6% of that response while reducing in-band interference by 6.4 dB. This processing test did not establish a usable mitigation. Table S3 and Supplementary Section S6 provide the settings and results.

### 3.6. Averaging Requirements and Impact Quality

The low-order shaft motion and the interference within the decision band are distinct quantities. The averaging estimate below uses the measured in-band margin, not the energy of the low-frequency rotation orders.

Scaling the stationary response by impact force gives a rotating in-band floor between 12.4 dB below and 4.1 dB above the expected response. Under the averaging model, placing the floor 10 dB below that response requires 1–26 averages, with a median of four [45]. This estimate is not an experiment with additional impacts and does not establish that the available six to eight impacts are sufficient. Figure S4 and Supplementary Section S7 report the impact-phase check used to assess the model [46].

Using only impacts that satisfy the quality criterion leaves at least three impacts in four rotating conditions. Their pre-impact comparison outcomes are unchanged, but candidate frequencies shift from 244.141 to 246.582 Hz at 100 rpm P3 and from 236.816 to 227.051 Hz at 200 rpm P3. Both P4 frequencies remain unchanged. The other four conditions retain only one or two clean impacts and are classified as untestable, not as agreement. Tables S2 and S6 report the band-maximum and recomputed candidate-frequency comparisons, respectively.

Leave-one-out analysis omitted each impact in turn and recomputed the remaining records, producing 58 analyses across eight conditions. Segmentation, delay fitting, spectral estimation, candidate detection and phase-trend testing were repeated for NCC. Image registration and preprocessing remained fixed. Of 44 omissions from conditions with an original candidate, 36 redetected a candidate, and eight did not. At the original frequency, the pre-impact comparison changed in 2/44 omissions. Within the 36 redetections, the original-frequency and reselected-frequency comparisons each changed once, in different omissions. At 100 rpm P4, all seven omissions returned a candidate, but only five reselected the original frequency. At 200 rpm P3, four of seven omissions redetected a candidate and two of those four retained one after both trend fits. The full-dataset phase-trend rejection is therefore sensitive to individual impacts. These overlapping subsets are not independent trials or a comprehensive statistical uncertainty assessment. Table S7 summarizes the condition-specific leave-one-out results. Supplementary Section S10 defines the detailed data files. Supplementary Figure S12 summarizes clean-impact counts, candidate redetection and phase-trend retention across the rotating conditions.

## 4. Discussion

The stationary optical–contact comparison supports identification of the first-mode region under the tested stationary conditions. P2 provides the stronger comparison because the P3 contact peak is broader, and the P3 optical phase criterion depends on the trend-fitting interval. This evidence does not validate the rotating optical measurements.

The broadband evidence in Section 3.4 and the processing checks in Supplementary Sections S5–S7 and S13 distinguish observed degradation from demonstrated mitigation. Neither the order-removal tests nor the averaging estimate establishes that a particular processing change restores modal identification.

The pre-impact control adds evidence from a segment that cannot contain the response to the target impact. Where a sufficiently long segment is available, the same recording can support a matched comparison without an additional sensor. Reference comparisons and consistency-based identification address complementary questions [15,24,26]. The control assesses whether coherence is sufficient evidence for an impact-related interpretation. It does not replace an independent shaft-response reference.

Tracker disagreement limits confidence in a unique modal frequency. Conversely, agreement between trackers is a processing-consistency check, not independent validation, because they share the optical measurement source. The tracker and the impacts included in each estimate should therefore be reported. Table S1 provides this information for the rotating measurements.

Improved illumination, optimized exposure, changes in frame rate and alternative optical configurations are potential mitigation strategies. They were not tested here. Controlled experiments should assess speckle correlation, noise floor and agreement with an independent reference to determine whether improved image quality produces more reliable modal identification. Independent non-contact shaft-response measurements were unavailable for this revision, leaving the rotating candidates unconfirmed.

## 5. Conclusions

The framework combines stationary optical–contact comparison using the same impacts, a matched pre-impact control during rotation, and assessment of optical degradation through the noise floor and speckle correlation. This experiment showed that plausible optical FRFs and high coherence were insufficient to establish modal identification during rotation.

A matched pre-impact control should accompany coherence when assessing optical modal identification under rotation. When a sufficiently long pre-impact segment is available, it can be processed with the same window length and procedure as the impact-response segment. Comparing coherence at the candidate frequency then tests whether it exceeds the pre-impact value. Coherence alone cannot validate the identification, and candidates not disqualified by this check still require independent validation.

The findings apply to one shaft, one optical configuration and two rotational speeds. Limited clean-impact counts and sensitivity to individual omissions constrain the conclusions. The fixed band may exclude shifted modes, and the neighboring pole may affect candidate interpretation and damping estimates. No additional independent trials, alternative-shaft experiments, optical mitigation experiments or independent non-contact reference measurements were performed. Broader applicability requires independent datasets, different geometries and materials, higher speeds and reference measurements of the rotating shaft response. Controlled optical tests are also needed to evaluate mitigation.

## Figures and Tables

**Table 1:** First bending mode at 0 rpm, from the same physical impacts recorded by the two independently triggered systems with the accelerometer on the shaft at x = 210 mm. Frequencies are identified by the phase criterion of Section 2.5 and damping from the half-power bandwidth of the same averaged estimate, with the exponential-window contribution removed. Coherence is quoted at the identified frequency of each channel, not as the maximum inside the band, which at 0 rpm falls at the lower band edge and does not describe coherence at the identified modal frequency. The frequency resolution, which bounds the resolvable difference, is 1.221 Hz optically and 0.625 Hz on the contact channel.

Position	Shots	Accelerometer *f*_r_ [Hz]	*ζ* [%]	*γ* ^2^	Optical *f*_r_ [Hz]	*ζ* [%]	*γ* ^2^	Difference [%]
P2 (*x* = 260 mm)	5	236.875	4.53	0.981	234.375	6.84	0.840	−1.06
P3 (*x* = 160 mm)	4	241.875	6.11	0.983	235.596	7.54	0.990	−2.60

**Table 2:** Contact accelerometer on the bearing housing, identified by the criterion of Section 2.5 and with damping from the half-power bandwidth. A dash means the criterion was not met inside 225–247 Hz. Each rotating condition includes eight impacts. The 0 rpm housing baseline has four shots per position except P3, which has eight, and no shot was discarded. Changes with speed are taken within each position against the value of that same position in the first column.

Position	0 rpm *f*_r_ [Hz]	*ζ* [%]	100 rpm *f*_r_ [Hz]	*ζ* [%]	200 rpm *f*_r_ [Hz]	*ζ* [%]
P1 (*x* = 320 mm)	234.375	4.68	–	–	–	–
P2 (*x* = 260 mm)	235.000	3.47	235.625	13.77	–	–
P3 (*x* = 160 mm)	236.250	4.04	233.125	7.61	226.875	8.30
P4 (*x* = 100 mm)	236.250	3.26	228.750	7.02	–	–

**Table 3:** Candidate detection, pre-impact comparison and phase-trend results for all eight rotating speed–position combinations using NCC and the full dataset. The selected frequency f* is reported when a candidate is detected. “Not disqualified” means impact-response coherence exceeds pre-impact coherence at f*. “Retained” means the same candidate is returned after trend removal under both fitting intervals. Neither result independently confirms a structural mode. A dash indicates no candidate and an unavailable comparison. Table S9 reports the coherence values and the separate band-maximum comparison.

Speed (rpm)	Pos.	Candidate f* (Hz)	Pre-Impact Control	Phase-Trend Test
100	P1	234.38	Disqualified	Retained
100	P2	None	—	—
100	P3	244.14	Disqualified	Retained
100	P4	227.05	Not disqualified	Retained
200	P1	None	—	—
200	P2	231.93	Disqualified	Retained
200	P3	236.82	Not disqualified	Not retained
200	P4	229.49	Not disqualified	Retained

## Data Availability

The data supporting the findings of this study are available from the corresponding author upon reasonable request.

## Supplementary Materials

The following supporting information can be downloaded at: https://www.mdpi.com/article/10.3390/s26217001/s1. Figure S1: Full-band stationary optical and accelerometer FRFs and coherence; Figure S2: Full-band rotating optical FRFs using phase correlation; Figure S3: Optical response spectra and rotation-order distributions; Figure S4: Impact-phase distribution and rotation-order suppression; Figure S5: Impact-force quality and force spectra; Figure S6: Stationary optical–contact comparison using both acquisition runs; Figure S7: Stationary FRF magnitude, phase, and phase-change criterion; Figure S8: Rotating optical FRF magnitude and phase-change criterion; Figure S9: Recorded speckle pattern, tracking ROI, and autocorrelation; Figure S10: Phase-change sensitivity to linear-trend removal; Figure S11: Candidate-detection sensitivity to analysis settings; Figure S12: Clean-impact and leave-one-out sensitivity analyses. Table S1: Tracker-specific rotating candidates and pre-impact comparisons; Table S2: Band-maximum coherence using all and clean impacts; Table S3: Vold–Kalman filtering null-test results; Table S4: Stationary identification at all four excitation positions; Table S5: Phase-trend sensitivity results; Table S6: Candidate detection and pre-impact comparison using clean impacts; Table S7: Leave-one-out sensitivity by measurement condition; Table S8: Sensitivity to the residual-delay fitting band; Table S9: NCC pre-impact coherence comparisons. Supplementary Sections S1–S14 provide additional methodological details and analyses. Separate numerical data files contain the leave-one-out and parameter-sensitivity results.

## Abbreviations

The following abbreviations are used in this manuscript:

FRF	frequency response function
NCC	normalized cross-correlation
PHCORR	phase correlation
PSD	power spectral density
ROI	region of interest
SKPCC	phase correlation with subpixel refinement
DAQ	data acquisition
LOO	leave-one-out
FDM	fused deposition modeling
ASA	acrylonitrile styrene acrylate
