# Supplementary material for: Validity Limits of High-Speed Laser-Speckle Modal Measurement on a Rotating Shaft: A Pre-Impact Falsification Test
Source: Sensors (Basel). 2026 Nov 5;26(21):7001. doi: 10.3390/s26217001 (PMC13766521; doi:10.3390/s26217001)
Supplement: Supplementary file 1 [file sensors-26-07001-s001.zip › sensors-4614735-supplementary.pdf]

sensors

MDPI

Article

Supplementary Material for: Validity Limits of High-Speed Laser-Speckle Modal Measurement on a Rotating Shaft: A Pre-Impact Falsification Test

Jongyun Kim [1], Seongkeol Kim [2,*]

[1] Department of Mechanical Design and Robot Engineering, Seoul National University of Science and Technology, Seoul 01811, Republic of Korea; 24520007@seoultech.ac.kr
[2] School of Mechanical Systems Engineering, Seoul National University of Science and Technology, Seoul 01811, Republic of Korea; rhett@seoultech.ac.kr
* Correspondence: rhett@seoultech.ac.kr

This supplement provides spectra, tracker candidates, impact-quality analyses, phase-trend and delay-fit sensitivity tests, and instrument and processing details. Numerical files accompanying this document contain the parameter-sensitivity and leave-one-out results.

The abbreviations used are normalized cross-correlation (NCC), phase correlation (PHCORR), phase correlation with subpixel refinement (SKPCC), frequency response function (FRF), region of interest (ROI), power spectral density (PSD) and leave-one-out (LOO). The ROI is 184 × 184 pixels. The reported decision band is 225–247 Hz. Trend-fitting intervals A and B are defined in Section S9. They are distinct from the original-frequency and reselected-frequency LOO comparisons defined in Section S10.

S1. Full-Band View at 0 rpm

Figure S1 extends the stationary comparison in Figure 3 of the main text to 0–2000 Hz using the same impacts and estimator. The optical response decreases above approximately 300 Hz. Additional contact-channel resonances occur at 733 and 847 Hz, outside the first-mode region considered here.

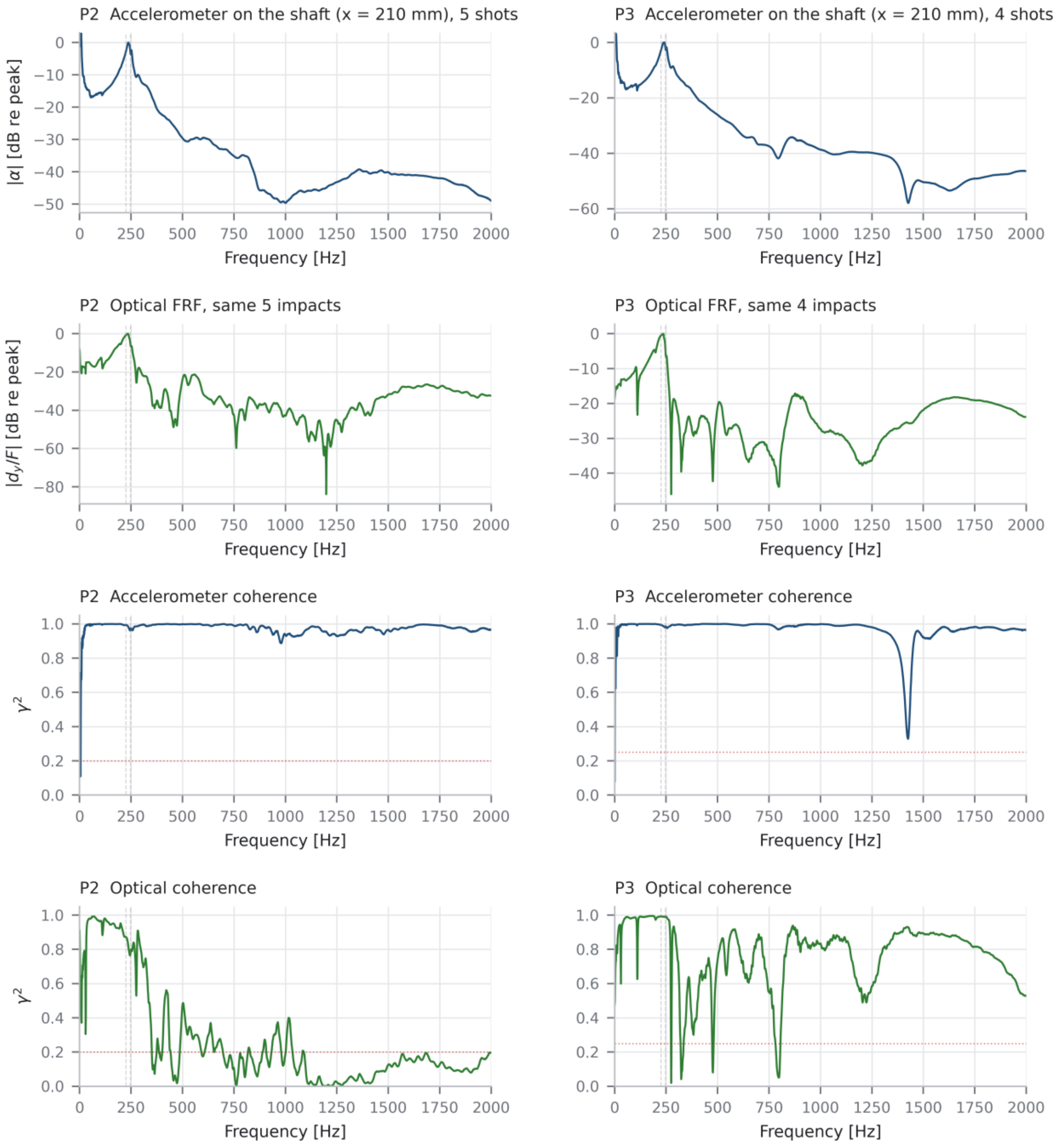

Figure S1. **At 0 rpm, P2 (left) and P3 (right), over the full analysis band 0–2000 Hz, from the same physical impacts recorded by the two independently triggered systems. Rows from top: receptance of the accelerometer mounted on the shaft at x = 210 mm, optical frequency response function (FRF), accelerometer coherence, optical coherence. Dashed vertical lines bound the decision band, 225–247 Hz. The dotted horizontal line on each coherence panel is the approximate null expectation 1/N for the number of averages used in that panel.**

S2. Full-Band View under Rotation

Figure S2 shows the PHCORR results at P2 and P3 at both rotating speeds over 0–2000 Hz. These panels place the decision band in the context of the full spectrum.

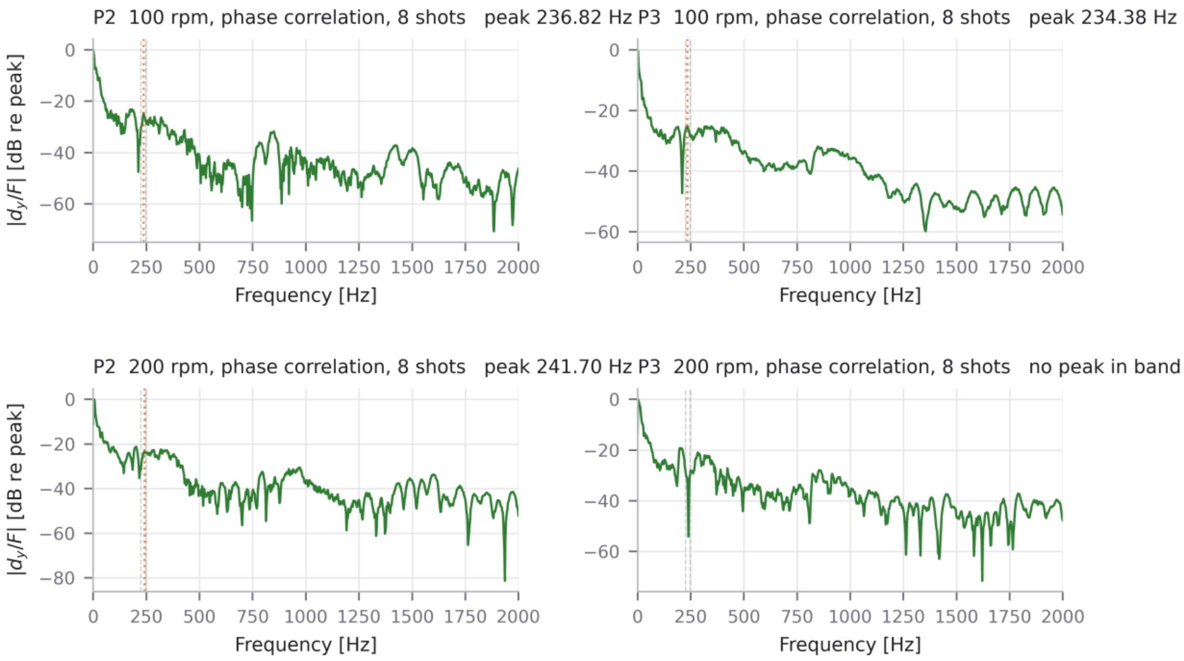

Figure S2. Optical FRF under rotation with the phase-correlation tracker, P2 (left) and P3 (right), 100 rpm (top) and 200 rpm (bottom), over the full analysis band 0–2000 Hz.

S3. Tracker Candidates and the Pre-Impact Control

Table S1 reports all detected rotating candidates and includes impact counts for the three trackers. Its coherence values are evaluated at the selected candidate frequency. Table S9 separately reports the band-maximum and candidate-frequency comparisons for NCC. Impact and pre-impact windows use identical impact sets within each tracking analysis, but the included sets may differ between trackers.

PHCORR candidates are not disqualified by the pre-impact comparison at 100 rpm P1, P2, and P4 and at 200 rpm P4. This does not independently establish structural modes. SKPCC was available only at 100 rpm, and no pre-impact comparison was performed for it.

Table S1. Rotating candidates and pre-impact comparisons by tracker. Condition gives speed in rpm and excitation position. N is the number of impacts. All detected candidates are listed, while coherence is compared at the selected frequency f*. A dash indicates no candidate or no available comparison. SKPCC was evaluated only at 100 rpm and did not undergo the pre-impact comparison.

Condition	Tracker	N	Candidates [Hz]	At f*, impact	At f*, control	Verdict
100 / P1	NCC	6	229.49, 234.38, 241.70	0.035	0.162	control higher
100 / P2	NCC	7	–	–	–	no candidate
100 / P3	NCC	8	244.14	0.210	0.752	control higher
100 / P4	NCC	7	227.05	0.583	0.045	impact higher
200 / P1	NCC	7	–	–	–	no candidate
200 / P2	NCC	8	231.93	0.077	0.282	control higher
200 / P3	NCC	7	236.82	0.577	0.435	impact higher
200 / P4	NCC	8	229.49	0.513	0.341	impact higher
100 / P1	PHCORR	6	231.93	0.793	0.661	impact higher
100 / P2	PHCORR	7	227.05, 236.82	0.238	0.067	impact higher
100 / P3	PHCORR	8	229.49, 244.14	0.172	0.509	control higher
100 / P4	PHCORR	6	231.93, 244.14	0.030	0.002	impact higher
200 / P1	PHCORR	7	246.58	0.240	0.471	control higher
200 / P2	PHCORR	8	–	–	–	no candidate
200 / P3	PHCORR	7	231.93	0.111	0.363	control higher
200 / P4	PHCORR	8	241.70	0.596	0.143	impact higher
100 / P1	SKPCC	6	229.49	–	–	candidate only
100 / P2	SKPCC	7	–	–	–	no candidate
100 / P3	SKPCC	8	–	–	–	no candidate
100 / P4	SKPCC	7	229.49, 246.58	–	–	candidate only

S4. Impact-Quality Sensitivity Analysis

The impact-quality criterion flags a secondary-force ratio of 0.20 or more, as defined in Section 2.7 of the main text. The clean-impact counts are given in Tables S2 and S6. The largest rotating ratio is 0.64 at 200 rpm at P3. For the stationary P2 and P3 comparisons, only two clean impacts remain at each position. The corresponding contact and optical frequencies are 236.875 and 233.154 Hz at P2 and 242.500 and 236.816 Hz at P3. These are within one spectral line of the reported estimates, but the small samples do not support a reliable clean-impact coherence comparison.

Table S2 repeats the band-maximum comparison using clean impacts. Four conditions have at least three clean impacts and retain the same comparison outcome. Four have only one or two and are classified as untestable. Table S6 separately recomputes candidate detection and the comparison at the selected frequency.

The main results retain all usable impacts. Figure S5 illustrates the quality criterion and the measured force spectra. Clean-impact outcomes are reported separately rather than replacing the original averages.

Across 75 impacts, the median force magnitude in the decision band is 6.5 dB below each impact's own maximum, with a 10th–90th percentile range of 8.6–3.4 dB below it. The corresponding medians are 6.3 dB at 0 rpm, 6.9 dB at 100 rpm and 6.1 dB at 200 rpm. The median remains within 11.2 dB of the maximum over 20–2000 Hz. The 5120 Hz force record is band-limited and does not resolve the true contact-force peak. Figure S5(c) therefore characterizes the excitation available to the estimator.

Table S2. Band-maximum coherence using all impacts and clean impacts with a secondary-force ratio below 0.20. Condition gives speed in rpm and position. Each numerical pair lists impact-response / pre-impact coherence. N lists all / clean impact counts. Fewer than three clean impacts are not tested. Candidate-frequency comparisons are reported separately in Table S6.

**Condition**	**N, all / clean**	**All: impact / control**	**All: verdict**	**Clean: impact / control**	**Clean: verdict**
100 / P1	6 / 2	0.087 / 0.618	control higher	– / –	not testable
100 / P2	7 / 2	0.872 / 0.518	impact higher	– / –	not testable
100 / P3	8 / 5	0.645 / 0.824	control higher	0.278 / 0.824	control higher
100 / P4	7 / 4	0.583 / 0.326	impact higher	0.814 / 0.558	impact higher
200 / P1	7 / 1	0.492 / 0.607	control higher	– / –	not testable
200 / P2	8 / 2	0.364 / 0.396	control higher	– / –	not testable
200 / P3	7 / 3	0.695 / 0.435	impact higher	0.621 / 0.512	impact higher
200 / P4	8 / 7	0.536 / 0.341	impact higher	0.572 / 0.346	impact higher

S5. Why the Rotation Orders Cannot Be Removed

The nominal rotating conditions are labeled 100 and 200 rpm throughout. The order calculations use rotational frequencies of 1.695 and 3.332 Hz, respectively, obtained from the motor-controller delay calibration. Two order-removal routes were evaluated before the Vold–Kalman test in Section S6. The first is frequency-domain separation. At 100 rpm, the shaft rotates at 1.695 Hz, so the orders falling inside 225–247 Hz are orders 133 to 145, thirteen of them, while the band contains only nine spectral lines at the attainable resolution of 2.441 Hz. Every line in the band lies on an order, and the band is fully occupied. At 200 rpm, the spacing is 3.332 Hz, which is wider than the 2.441 Hz resolution, and the occupancy is 78 %. Using a conservative criterion that sets the Fourier bin spacing to no more than half the 100 rpm order spacing ($\Delta f \leq 0.85$ Hz), resolving the orders would require approximately 23,530 samples, essentially the whole record, whereas the response has decayed within 40 ms. The binding constraint is therefore the length of the window over which the transient response exists, not the length of the record, which spans about two revolutions at 100 rpm and four at 200 rpm. Figure S3 shows the optical response spectrum at 0 rpm and at 200 rpm with the in-band orders marked.

The second is time-domain synchronous subtraction, which is limited by the number of impact-free revolutions available before the impact. The record spans 2.01 revolutions at 100 rpm and 3.95 at 200 rpm, with 0.97 and 1.94 revolutions respectively before the impact, so at 100 rpm there is not a full revolution of impact-free signal to subtract. Subtracting the same-angle segment one revolution away reduces the post-impact variance

by 44 to 49 % at 200 rpm, but the effect on the coherence is almost entirely a collapse of the control window rather than an improvement of the impact window, which is consistent with the removed component being deterministic runout that both windows share.

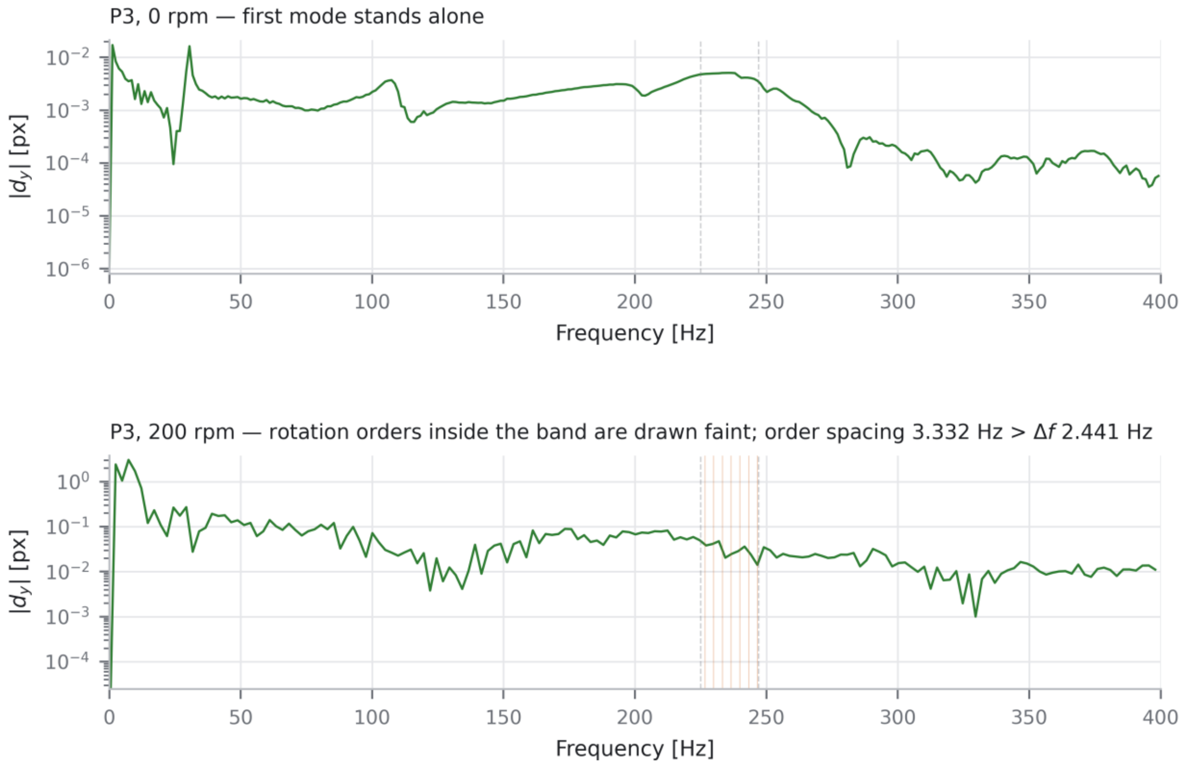

Figure S3. Optical response spectrum at P3 in pixels, at 0 rpm (top) and at 200 rpm (bottom), with no window applied. Faint vertical lines in the lower panel mark the rotation orders that fall inside the decision band. The in-band amplitude rises by roughly an order of magnitude, and the band is densely populated by rotation orders**.** At 200 rpm, the order spacing of 3.332 Hz exceeds the 2.441 Hz resolution, and 78 % of the lines in the band lie on an order, while at 100 rpm, the spacing of 1.695 Hz is smaller than the resolution and every spectral line in the decision band is occupied by a rotation order.

S6. Vold–Kalman Null Test

Because Vold–Kalman order filtering operates in the time domain under a smoothness constraint on the order envelopes, it is not bounded by the Fourier resolution that blocks frequency-domain order removal. The multi-order first-generation filter was implemented and tested. The data equation requires the sum of the order components to reproduce the measurement, and the structural equation requires the second difference of each complex envelope to be small, weighted by a factor r. The two are solved together as one weighted least-squares problem, and the fitted order content is reconstructed at the original sample rate and subtracted in the time domain, so that no filter is applied to the analyzed signal. After heterodyning to the band center, the complex order envelopes were decimated by a factor of 100 and solved on a 200 Hz envelope grid. The fit is therefore carried out on that decimated grid and not at the acquisition sample rate.

The test is a null test. A synthetic decaying sinusoid at 236 Hz with 5 % damping was added to a measured pre-impact segment that contains rotation-borne content but no response, with an amplitude of 0.4 times the standard deviation of that segment; the order removal was applied, and the fraction of the synthetic response surviving was measured together with the reduction of in-band interference. The survival fractions reported below

are therefore conditional on this synthetic-to-interference level and should not be read as universal Vold–Kalman performance limits. Table S3 gives the outcome over the configurations tested.

The observed limitation is consistent with the time-scale mismatch inherent in the present transient-response problem. The separating power of the filter comes from the assumption that the order envelopes vary slowly compared with the order spacing. Separating orders spaced 3.332 Hz apart calls for an envelope whose characteristic timescale is of order 1/3.332 = 300 ms, whereas the response decays with a time constant of $1/(2\pi f_r \zeta)$ = 13.5 ms, some twenty-two times faster. At 100 rpm, the corresponding timescale is 590 ms, and the ratio is about forty-four. A rapidly decaying transient whose frequency coincides with an order is therefore indistinguishable, within the model, from an order whose envelope happens to vary quickly, and the filter absorbs it. The same conclusion follows from a degrees-of-freedom count: a 0.38 s segment represented on that 200 Hz envelope grid provides approximately 150 real data points against 1050 to 5400 unknowns, so the solution is determined almost entirely by the smoothness constraint, which is precisely the property the response violates.

Table S3. Vold–Kalman multi-order null test. The first four rows are at 200 rpm and the last at 100 rpm, both at P3. A synthetic decaying sinusoid at 236 Hz with 5 % damping was added to a measured pre-impact segment. Survival is the fraction of that synthetic response remaining after order removal**.** The in-band change is that of the same segment without the synthetic component.

Order set	Weight r	Synthetic response survival [%]	In-band change [dB]
7 orders inside 225–247 Hz	1e1	14.1	−6.93
7 orders inside 225–247 Hz	1e5	18.6	−6.42
36 orders, 176–296 Hz	1e0	4.4	−11.16
36 orders, 176–296 Hz	1e3	8.4	−5.52
71 orders, 176–296 Hz (100 rpm)	1e4	7.9	−1.27

S7. Impact Phase and Impact Quality

Figure S4 reports the estimated impact phases and rotation-order suppression at 200 rpm P3. The low-order suppression is 0.35, close to the random-phase estimate $1/\sqrt{8}$ = 0.354. In-band values range from 0.077 to 0.621. This is a consistency check on the averaging model, not proof that the available impact counts are sufficient.

Phase-controlled impacts could suppress selected rotation orders, but orders whose indices are multiples of the number of uniformly spaced phases would remain. Any implementation would require an appropriate phase count and independent testing.

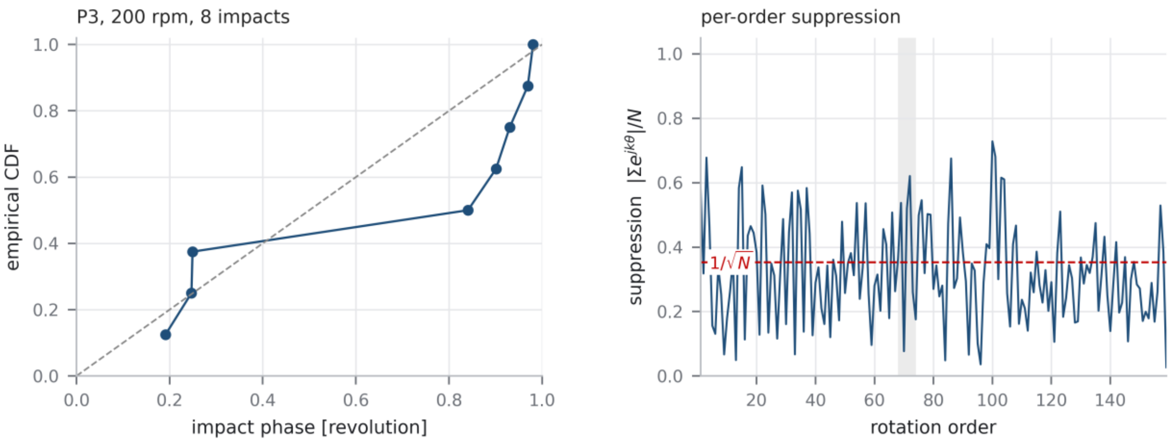

Figure S4. Impact phase at 200 rpm, P3. Left: empirical distribution of the shaft phase at the instant of impact, against a uniform set. Right: the resulting suppression of each rotation order by impact-synchronous averaging**.** The dashed line indicates the theoretical $1/\sqrt{N}$ suppression expected for random-phase averaging, and the shaded band marks the orders inside 225–247 Hz.

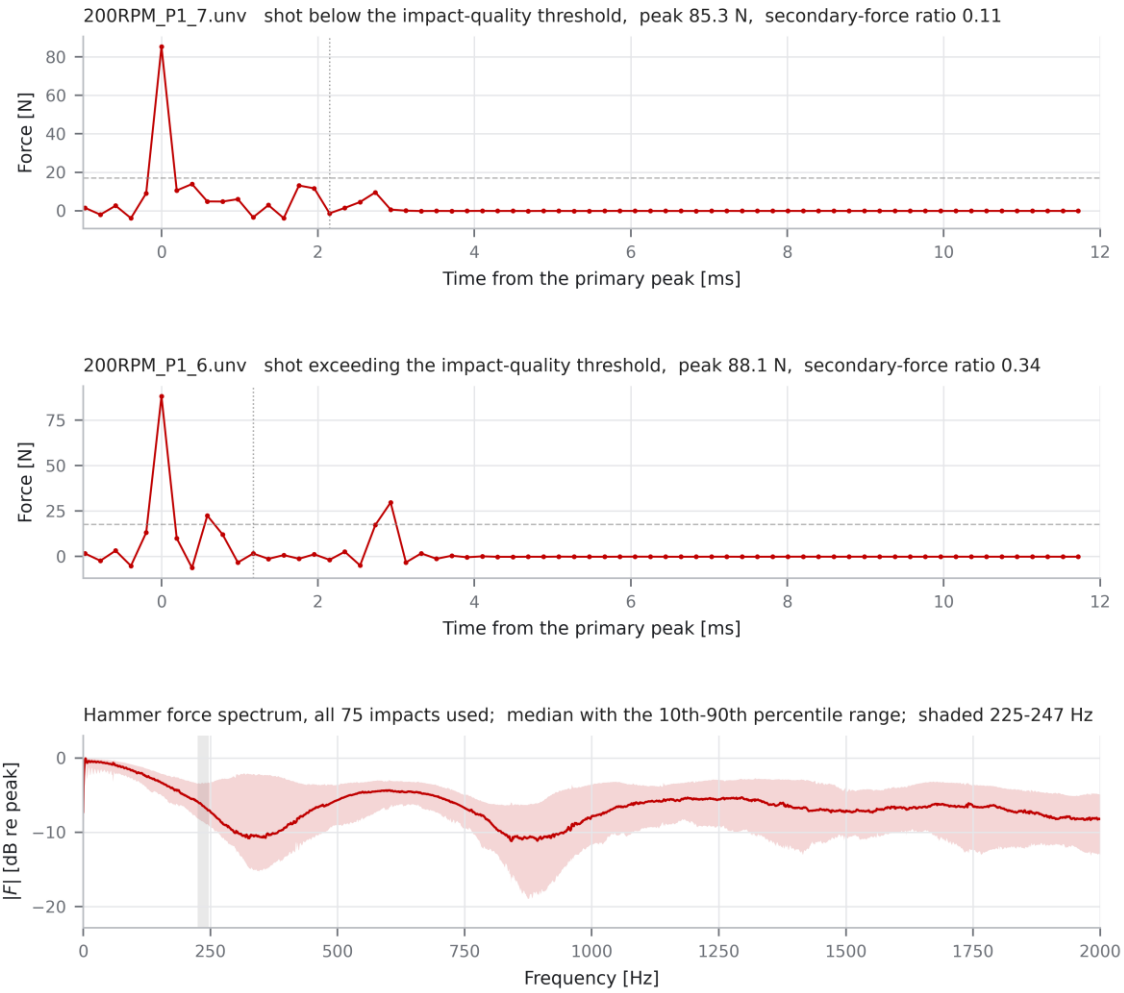

Figure S5. Measured impact force. (a) A shot below the impact-quality threshold at 200 rpm and P1, secondary-force ratio 0.11. (b) A shot from the same measurement condition exceeding the threshold, secondary-force ratio 0.34, with a later force peak 2.9 ms after the primary peak. Panels (a) and (b) show the acquired samples of the force channel at 5120 Hz, the grid on which the criterion of Section 2.7 of the main text is evaluated. The dashed line is 20 % of the primary peak, and the dotted line marks the sample at which the primary pulse first falls below 2 % of that peak, after which the ten-millisecond evaluation window begins. (c) Magnitude spectrum of the hammer force for all 75 impacts entering the reported analyses at 0, 100 and 200 rpm, each normalized to its own maximum. The solid curve is the median over impacts, and the band is the 10th to 90th percentile range. The shaded strip is the 225–247 Hz decision band.

S8. Stationary Results at All Four Positions and over Both Acquisition Runs

Table S4 gives all four stationary positions from the second acquisition run. Both channels return a candidate only at P2 and P3. At P1, only the optical channel returns a candidate. At P4, only the contact channel does. The paired frequency comparison in Table 1 is therefore limited to P2 and P3.

Figure S6 pools both acquisition runs, giving nine impacts at P2 and eight at P3. All four identified frequencies remain unchanged. Contact coherence changes from 0.981 to 0.963 at P2 and from 0.983 to 0.981 at P3. Optical coherence decreases from 0.840 to 0.699 and from 0.990 to 0.299, respectively. The main analysis uses the second run because the illuminated spot occupied a consistent sensor position across excitation positions.

Table S4. Stationary result at all four excitation positions, second acquisition run, both channels, by the criterion of Section 2.5 of the main text. A dash means the criterion returned no candidate inside 225–247 Hz. Damping is from the half-power bandwidth with the exponential-window contribution removed, and coherence is quoted at the identified frequency. P2 and P3 are the two positions at which both channels return a candidate**.** They are the rows reported in Table 1 of the main text.

**Position**	**Shots**	**Contact f_r [Hz]**	**ζ [%]**	**γ^2**	**Optical f_r [Hz]**	**ζ [%]**	**γ^2**	**Difference [%]**
P1 (x = 320 mm)	4	–	–	–	236.816	6.58	0.814	–
P2 (x = 260 mm)	5	236.875	4.53	0.981	234.375	6.84	0.840	−1.06
P3 (x = 160 mm)	4	241.875	6.11	0.983	235.596	7.54	0.990	−2.60
P4 (x = 100 mm)	5	236.875	5.61	0.981	–	–	–	–

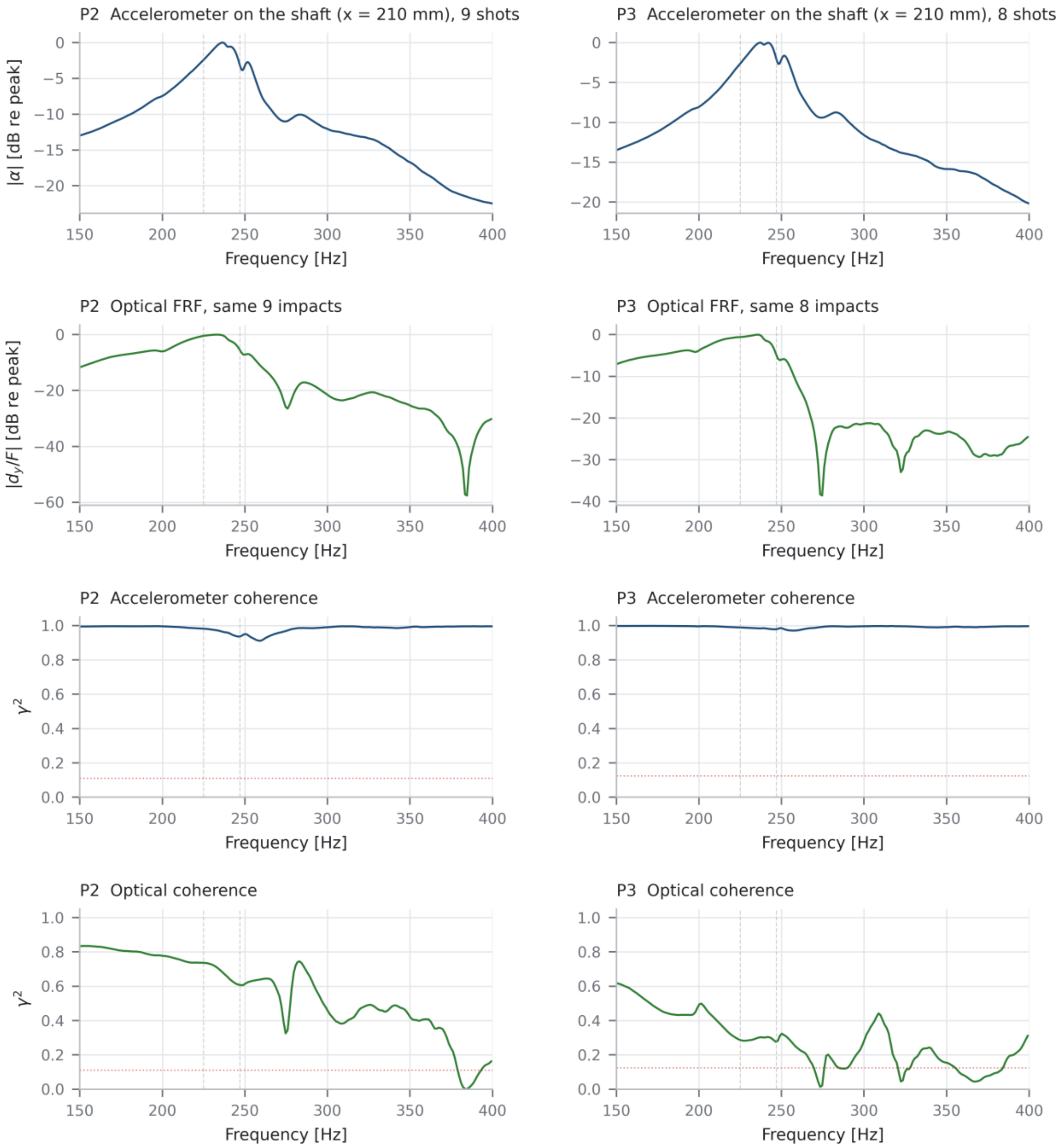

Figure S6. At 0 rpm over 150–400 Hz, P2 (left) and P3 (right), pooling both acquisition runs: nine shots at P2 and eight at P3, against the five and four in Figure 3 of the main text. Rows and axes are those of Figure 3.

S9. Phase of the Frequency Response Function Used by the Identification Criterion

Figures S7 and S8 show FRF magnitude, unwrapped phase and signed windowed phase decrease R(f) from the same averaged estimates as the reported candidates. The solid vertical line marks the candidate frequency at a magnitude maximum. The dashed line marks the associated phase-change maximum. They need not coincide because the criterion permits a separation of four spectral lines. The shaded interval is the phase window centered on that phase-change maximum. The bottom-row horizontal lines show the 45° threshold and the background median.

Figure S8 illustrates three NCC outcomes at 100 rpm: no candidate at P2, a candidate disqualified by the pre-impact comparison at P3, and a candidate not disqualified at P4. The P2 spectrum also illustrates why high band-maximum coherence alone is insufficient.

Stationary phase-window half-widths of 6, 8, 10, 12, 14, 16, 18 and 20 Hz were tested. Detected frequencies remained 234.375 and 236.875 Hz at P2 and 235.596 and 241.875 Hz at P3 for optical and contact channels, respectively. Optical candidates appeared at all settings. Contact candidates were absent at two settings at each position. Simple magnitude peak picking returned the same frequencies. For rotating sensitivity, all 36 combinations of thresholds 30°, 45° and 60°, half-widths 8, 10 and 12 Hz, and bands 225–247, 220–252, 230–242 and 215–257 Hz were evaluated. sensitivity_settings.csv gives one-setting-at-a-time comparisons. sensitivity_all_combinations.csv gives the full results, including candidate frequencies and pre-impact comparisons. Neither analysis establishes that a newly included candidate is a speed-shifted mode.

The signed phase criterion and linear-trend contribution are defined in Equations (6)–(8) of the main text. The background in Table S5 is a median of R(f), not the fitted linear-trend contribution. It must not be subtracted from the accepted value to infer the result after detrending.

Trend fits A (150–200 and 300–400 Hz) and B (120–200 and 300–450 Hz) exclude the candidate-search neighborhood. The fitted line is removed from the unwrapped phase before the unchanged detection rule is applied. A trial fit over 200–300 Hz excluding only 225–247 Hz also removed stationary contact candidates, indicating sensitivity to contamination of the fit by the resonance region. It is not used for the reported results. Table S5 reports both retained fitting intervals. The original candidate and control results remain separate from this diagnostic.

For NCC at 200 rpm P3, the original accepted phase-change maximum of 48.2° falls to 19.5° after fit A and 24.5° after fit B, evaluated at the same phase-change peak and discrete window. The other five selected rotating NCC candidates remain above 45° under both fits. PHCORR at 100 rpm P1 and its secondary candidate at 100 rpm P2 are not retained. The stationary P3 optical candidate remains only under fit B. These are full-dataset outcomes and do not imply stability to individual impact omissions.

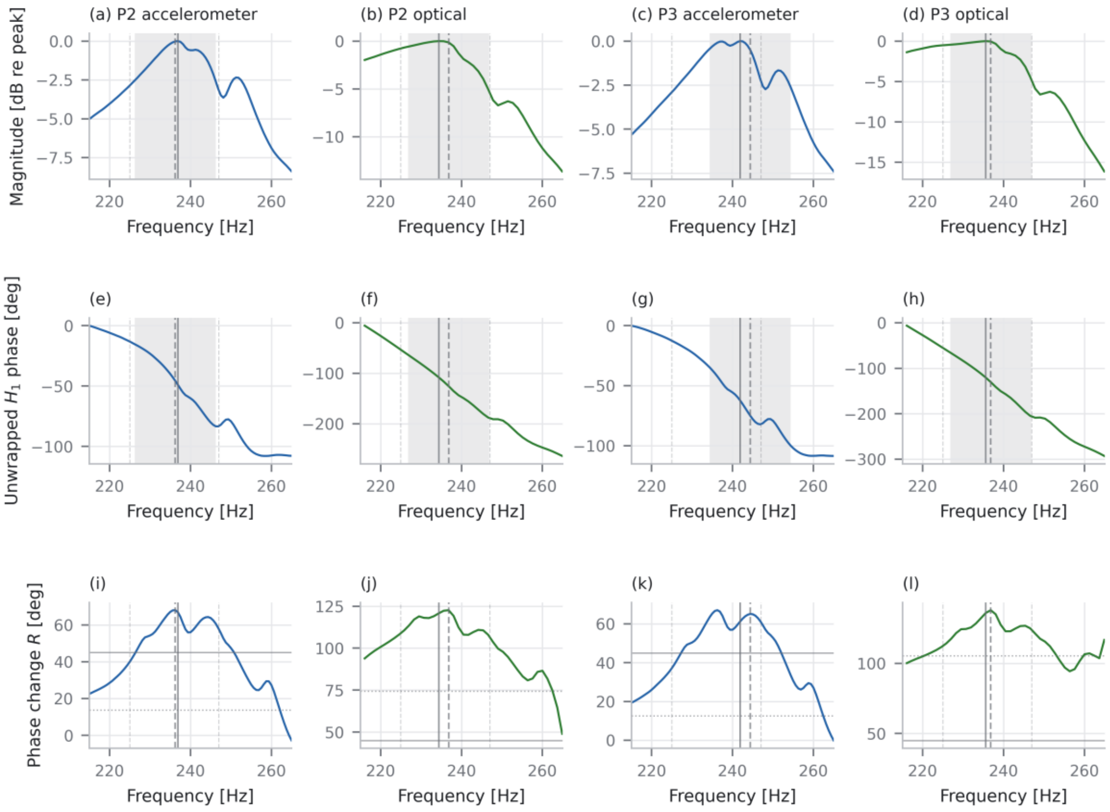

Figure S7. At 0 rpm, second acquisition run, over 215 to 265 Hz. Columns from left: P2 accelerometer, P2 optical, P3 accelerometer, P3 optical. **The top row sh**ows **the FRF** magnitude normalized to its maximum over the displayed interval**. The middle row shows** the unwrapped phase of the same complex H_1 estimate re**lativ**e to its value at the left edge**. The bottom row shows** the windowed phase change R(f). Markers are as described above. Faint dashed vertical lines bound the decision band, 225–247 Hz.

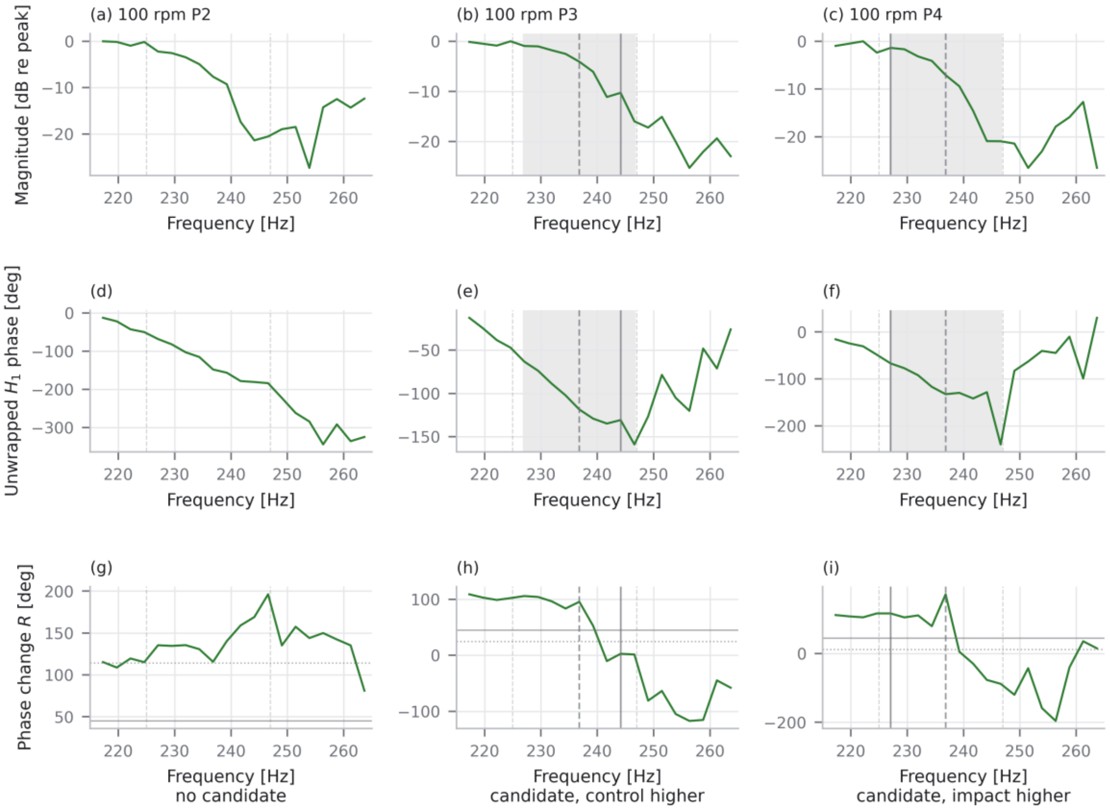

Figure S8. Three rotating measurement conditions at 100 rpm with the normalized cross-correlation tracker, over 215 to 265 Hz, with the rows, axes and markers of Figure S7. **The left c**olumn s**hows** P2, where the criterion returns no candidate**. The middle column shows** P3, where **the candidat**e **i**s 244.141 Hz, and the pre-impact cohe**rence is** higher at that **frequency. The right colum**n **shows** P4, where **the candidate i**s 227.051 Hz, and the impact**-res****ponse coherence is higher**. In the P4 column, the two vertical markers are 9.8 Hz apart, four spectral lines at this resolution.

Table S5. Phase-trend sensitivity test. The candidate frequency and the accepted phase change are those returned by the unmodified criterion of Section 2.5 of the main text. The background is the median of R(f) over 200 to 300 Hz, excluding ±15 Hz around the candidate. The last two columns state whether the same candidate is returned after a straight line fitted over interval A or interval B is subtracted from the unwrapped phase. Yes means the same candidate frequency is returned after the subtraction, and no means it is not. A dash means the criterion returns no candidate in that measurement condition, in which case the test cannot change the outcome.

Channel or tracker	Cell	Candidate [Hz]	Accepted R [deg]	Background [deg]	A	B
Contact, 0 rpm	P2	236.875	67.9	13.7	yes	yes
Contact, 0 rpm	P3	241.875	65.1	12.6	yes	yes
Optical, 0 rpm	P2	234.375	122.6	74.3	yes	yes
Optical, 0 rpm	P3	235.596	137.9	105.4	no	yes
NCC, 100 rpm	P1	234.375	208.1	34.1	yes	yes
NCC, 100 rpm	P2	–	–	114.3	–	–
NCC, 100 rpm	P3	244.141	95.7	24.9	yes	yes
NCC, 100 rpm	P4	227.051	172.7	12.5	yes	yes
NCC, 200 rpm	P1	–	–	18.9	–	–
NCC, 200 rpm	P2	231.934	153.0	43.5	yes	yes
NCC, 200 rpm	P3	236.816	48.2	58.3	no	no
NCC, 200 rpm	P4	229.492	127.8	45.3	yes	yes
PHCORR, 100 rpm	P1	231.934	45.3	−2.6	no	no
PHCORR, 100 rpm	P2	227.051	177.2	3.9	yes	yes
PHCORR, 100 rpm	P2 (second)	236.816	46.3	3.9	no	no
PHCORR, 100 rpm	P3	244.141	131.6	111.9	yes	yes
PHCORR, 100 rpm	P4	231.934	426.0	−73.8	yes	yes
PHCORR, 200 rpm	P1	246.582	92.5	−20.0	yes	yes
PHCORR, 200 rpm	P2	–	–	−3.0	–	–
PHCORR, 200 rpm	P3	231.934	97.1	11.9	yes	yes
PHCORR, 200 rpm	P4	241.699	66.7	−4.2	yes	yes

S10. Clean-Impact and Leave-One-Out Reanalysis

Table S6 recomputes the FRF, candidate detection and pre-impact comparison using only clean impacts. Conditions with fewer than three clean impacts are not evaluated. This differs from Table S2, which compares band maxima rather than the selected candidate frequency.

The pre-impact comparison outcome is unchanged in all four testable conditions. The candidate frequency shifts from 244.141 to 246.582 Hz at 100 rpm P3 and from 236.816 to

227.051 Hz at 200 rpm P3. The P4 frequencies are unchanged. These results do not establish that quality of impact has no effect.

Leave-one-out analysis omits each impact once, giving 58 overlapping subsets (6 + 7 + 8 + 7 + 7 + 8 + 7 + 8). Segmentation, delay fitting, H1 estimation, coherence, candidate detection and phase-trend tests are repeated from fixed NCC tracking outputs and force records. Table S7 summarizes the condition-specific results. LOO_summary.csv and LOO_per_omission.csv report original-frequency evaluation A and reselected-frequency evaluation B. Evaluation A can be calculated even when no candidate is redetected. Evaluation B requires a candidate. phase_trend_summary.csv and phase_trend_per_omission.csv use separate fitting-interval labels A and B, defined in Section S9. Of the 44 omissions from conditions with an original candidate, 36 redetect one. Among those, 32 retain candidates after trend fit A and 34 after fit B. Of the 37 omissions from the five conditions retained in the full-set phase test, 32 redetect a candidate, and five do not. The conditional retained counts are 30/32 and 32/32. Candidate loss is not counted as retention. These are sensitivity counts, not independent statistical trials.

Table S6. Candidate detection and pre-impact comparison using all impacts and clean impacts. Condition gives speed in rpm and position. N lists all / clean impact counts. Verdicts compare coherence at the selected candidate frequency f*. A dash denotes no candidate or an unavailable evaluation, as specified in the verdict column.

Condition	N, all / clean	All: f* [Hz]	All: verdict	Clean: f* [Hz]	Clean: verdict
100 / P1	6 / 2	234.375	control higher	-	not testable
100 / P2	7 / 2	-	no candidate	-	not testable
100 / P3	8 / 5	244.141	control higher	246.582	control higher
100 / P4	7 / 4	227.051	impact higher	227.051	impact higher
200 / P1	7 / 1	-	no candidate	-	not testable
200 / P2	8 / 2	231.934	control higher	-	not testable
200 / P3	7 / 3	236.816	impact higher	227.051	impact higher
200 / P4	8 / 7	229.492	impact higher	229.492	impact higher

Table S7. Leave-one-out sensitivity by condition. N is the number of omitted-impact subsets. “Found” gives subsets with a candidate / N. A and B denote fixed-frequency and reselected-frequency evaluations. Changes are relative to the full-set pre-impact verdict. Phase retention is conditional on candidate redetection and is shown for trend-fitting intervals A ∣ B, which are distinct from evaluations A ∣ B. A dash indicates an unavailable comparison. These overlapping subsets are not independent trials.

**Condition (rpm / P)**	**N**	**Found**	**Verdict changes Evaluation A / B**	**Same f* Evaluation B**	**Phase retained Fit A / B**
100 / P1	6	4/6	1/6 ∣ 0/4	1/4	4/4 ∣ 4/4
100 / P2	7	0/7	– ∣ –	–	— ∣ —
100 / P3	8	6/8	0/8 ∣ 0/6	6/6	5/6 ∣ 6/6
100 / P4	7	7/7	0/7 ∣ 0/7	5/7	7/7 ∣ 7/7
200 / P1	7	0/7	– ∣ –	–	— ∣ —
200 / P2	8	7/8	0/8 ∣ 1/7	4/7	6/7 ∣ 7/7
200 / P3	7	4/7	1/7 ∣ 0/4	2/4	2/4 ∣ 2/4
200 / P4	8	8/8	0/8 ∣ 0/8	8/8	8/8 ∣ 8/8

S11. Sensitivity to the Band Used for the Residual Delay Fit

Residual delay was refitted over 120–200 Hz and 300–450 Hz, and was also left uncorrected. All other settings and the included impacts were held fixed. The impact and pre-impact estimates received the same processing in each variant. Table S8 compares these results with the reported 200–300 Hz fit. Stationary cross-validation uses onset alignment without this residual-delay fit.

With the 120–200 Hz fit, six of eight outcomes and six candidate-frequency entries agree with the reported processing. Both P4 frequencies and comparison outcomes remain unchanged. However, 100 rpm P2 gains a candidate and 200 rpm P3 loses one, so unchanged aggregate counts do not mean unchanged condition-specific results.

With the 300–450 Hz fit, four outcomes agree, and 200 rpm P4 changes to control higher. Without a delay fit, five conditions return no candidate. These results demonstrate sensitivity to alignment choices. They do not isolate the cause of the 12.21 Hz difference between NCC and PHCORR, whose individual impact identities were not verified as identical.

Table S8. Sensitivity to the residual-delay fitting band. Condition gives speed in rpm and position. Entries give f* in Hz and the window with higher coherence at f*: impact or control. All other settings and impact sets remain fixed. "No candidate" means none was detected in 225–247 Hz.

Condition	200–300 Hz (reported)	120–200 Hz	300–450 Hz	No delay fit
100 / P1	234.38, control	234.38, control	239.26, control	239.26, control
100 / P2	no candidate	246.58, impact	no candidate	no candidate
100 / P3	244.14, control	244.14, control	no candidate	no candidate
100 / P4	227.05, impact	227.05, impact	231.93, impact	231.93, impact
200 / P1	no candidate	no candidate	no candidate	no candidate
200 / P2	231.93, control	231.93, control	227.05, impact	no candidate
200 / P3	236.82, impact	no candidate	no candidate	no candidate
200 / P4	229.49, impact	229.49, impact	239.26, control	241.70, impact

Table S9. NCC pre-impact comparisons. Condition gives speed in rpm and position, and N is the shared impact count. Band maxima are evaluated separately in each window and disqualify four of eight conditions. At f*, impact-response and pre-impact coherence are compared at the same frequency and disqualify three of six conditions with candidates. Both windows use the same impacts, length and processing. A dash means no candidate.

Condition	N	Band maximum: impact	Band maximum: control	f* [Hz]	At f*: impact / control	Verdict at f*
100 / P1	6	0.087 at 241.70 Hz	0.618 at 229.49 Hz	234.38	0.035 / 0.162	control higher
100 / P2	7	0.872 at 227.05 Hz	0.518 at 239.26 Hz	–	– / –	no candidate
100 / P3	8	0.645 at 231.93 Hz	0.824 at 246.58 Hz	244.14	0.210 / 0.752	control higher
100 / P4	7	0.583 at 227.05 Hz	0.326 at 241.70 Hz	227.05	0.583 / 0.045	impact higher
200 / P1	7	0.492 at 227.05 Hz	0.607 at 231.93 Hz	–	– / –	no candidate
200 / P2	8	0.364 at 244.14 Hz	0.396 at 244.14 Hz	231.93	0.077 / 0.282	control higher
200 / P3	7	0.695 at 244.14 Hz	0.435 at 236.82 Hz	236.82	0.577 / 0.435	impact higher
200 / P4	8	0.536 at 234.38 Hz	0.341 at 229.49 Hz	229.49	0.513 / 0.341	impact higher

S12. Instrumentation, Optical Configuration and Acquisition Details

Instrument settings and calibration details supplement Section 2.2 of the main text.

The impulse hammer was used throughout on the most sensitive of its three ranges, 100 mV/lbf, that is 22.48 mV/N, for which the full-scale force is 50 lbf or 222 N. The head mass is 150 g. The accelerometer has a nominal sensitivity of 100 mV/g with a tolerance of

10 %, a range of 50 g peak, and a response flat within 5 % from 0.5 to 10 kHz; a sensitivity correction factor of 1.018 was applied because the analyzer was configured with 101.8 mV/g against the nominal value. The acquisition block is 8192 time samples rather than a line count, which at 5120 Hz is a 1.6 s record of 0.625 Hz resolution and yields 3200 usable spectral lines up to the 2000 Hz analysis limit, with the trigger at sample 836 of every block. The stationary optical record is 4697 frames long, so a window of 8192 frames does not exist there, which is why the stationary optical analysis uses 4096 samples.

The grain diameter is 1.25 pixels, estimated from the autocorrelation of the high-pass-filtered illuminated surround, with clipped pixels excluded. The focused-image estimate of 1.22 λN at 532 nm and f/22 gives approximately 14.3 µm, or 1.4 pixels. Here λ is wavelength, and N is the lens f-number. This estimate is only an order-of-magnitude comparison for the defocused geometry. Figure S9 shows the recorded frame, ROI and autocorrelation.

Within the ROI, 7.2% of pixels reach the white level. They are not masked. The unclipped surround has an intensity contrast of 0.27. These properties may reduce tracking sensitivity. Lower exposure or weaker illumination could reduce clipping, but neither was tested. Fixed optical settings do not by themselves exclude interactions with surface motion or frame rate.

Camera headers report 5000 frames per second at 0 rpm and 20,000 during rotation. The stationary frequency comparison supports the former. For rotating records, the 20,000 frames-per-second hypothesis places the first three rotation orders at the expected frequencies and is favored over the alternative hypothesis by 83.7 dB at 100 rpm and 90.3 dB at 200 rpm.

Camera setup blocks agree apart from frame-rate fields, a derived frame-period value and a rate-dependent table. Exposure (30 µs), sensor format (256 × 256), gain and black level are unchanged. The 184 × 184 ROI, optical geometry and tracking parameters are fixed. The frame-rate difference remains a limitation. Section S13 tests increment accumulation, not re-acquisition at a different rate.

The nominal lever length L2 = 102 mm was not independently calibrated. Equations (1) and (2) of the main text therefore provide geometric equivalents only. All reported measurements remain in pixel units, and no angular conversion affects candidate-frequency identification.

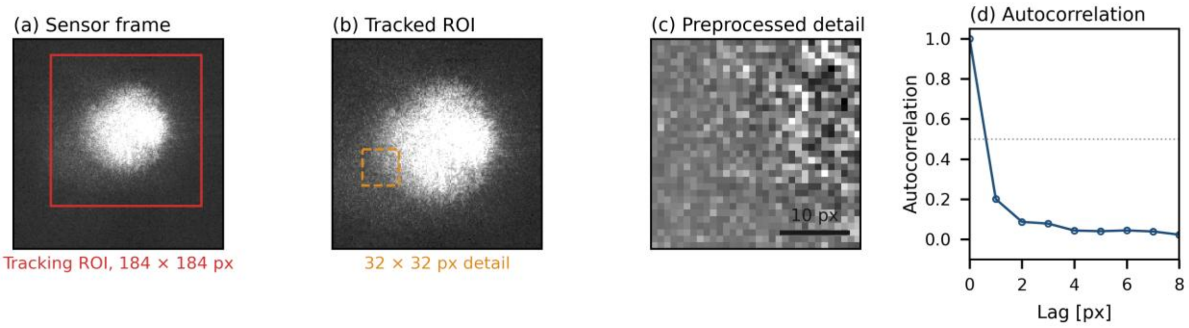

Figure S9. Speckle recorded in this experiment: the first frame of the stationary record at P3, second acquisition run, one of the records averaged in Table 1 of the main text. The tracking region drawn here is the one that record was tracked with. (a) the full 256 × 256 pixel sensor frame, with the 184 × 184 pixel tracking region outlined in red. (b) that tracking region, with the 32 × 32 pixel detail window outlined in orange. (c) the detail window after the tracker preprocessing of Section 2.3 of the main text, taken from the illuminated surround where no pixel is clipped. The tracker is applied to the whole region without masking, and panel (c) illustrates the usable texture. (d) the autocorrelation of the high-passed pattern over that surround, averaged over the two image axes, whose half-maximum width gives a grain diameter of 1.25 pixels against the 1.43 pixels predicted above. Panels (a) and (b) carry a gamma stretch so that the granular structure remains visible beside the bright core, which is clipped at the white level over 7.2 % of the tracking region.

S13. Signal-Processing Details and Processing Checks

This section gives processing checks supporting Sections 2.4 and 3.4 of the main text.

The mains-comb subtraction was tested on synthetic single-degree-of-freedom responses at 236 Hz generated from the measured force records and carrying no mains content, passed through the identical subtraction. The identified frequency moved by at most one spectral line, while the half-power damping ratio fell by 1.5 percentage points at an input damping of 3.0 %, 2.1 percentage points at 4.5 % and 3.3 percentage points at 7.0 %. Leaving the harmonic in place is worse: recomputing the measured accelerometer response functions with the fourth harmonic excluded from the comb moves the identified frequency by up to 8.75 Hz and the damping ratio by up to 5 percentage points. The subtraction is therefore retained, and the damping ratios reported in the main text carry a systematic uncertainty of a few percentage points from this operation alone.

The force record is resampled onto the camera time base with the exact integer ratio, 125/32 for 20,000 frames per second. The force onset is the last sample before the pulse rises above 2 % of its peak, and the optical onset is taken from the envelope of the 200–300 Hz band-pass of the trajectory, walking back from its maximum to 20 % of that maximum. Starting the window at the force maximum instead loses 0.59 ms of the rise, which costs 2.46 dB in magnitude and 51° in phase, which is why the window is positioned from the detected onset.

A force window was tested but did not change the reported condition-level outcomes and is not used. Response-only filtering would alter the FRF, while non-causal filtering could contaminate the pre-impact segment. The anti-aliasing requirements and the distinction between onset detection and analyzed signals are stated in Section 2.4 of the main text.

Measured frame-to-frame displacements are 0.277 pixels at 100 rpm and 0.628 pixels at 200 rpm. Relative to a 184-pixel ROI, rigid translation leaves overlap fractions of 0.997 and 0.993. The overlap loss is therefore much smaller than the observed correlation

decrease. After cumulative summation and linear detrending, trajectory excursions are 7.9–13.0 pixels.

Grouping rotating increments in fours changes the 400–700 Hz level by 0.0–0.2 dB. This tests accumulation of increments, not tracking between frames four apart. It cannot reproduce a recording acquired at a lower frame rate or isolate exposure effects.

S14. Additional Visual Summaries of Sensitivity Analyses

Figures S10–S12 provide visual summaries of reviewer-requested sensitivity analyses based entirely on the existing dataset. Figure S10 illustrates the phase-trend check described in Section S9, Figure S11 shows candidate-detection sensitivity to selection criteria discussed in Section S9, and Figure S12 summarizes clean-impact and leave-one-out results detailed in Sections S4 and S10.

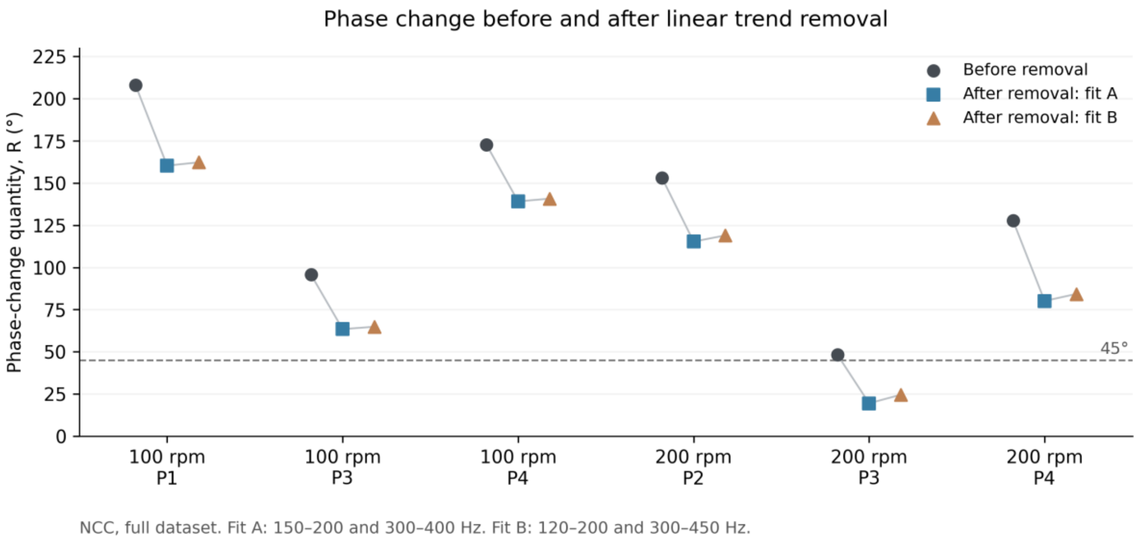

Figure S10. Phase-change quantity before and after linear trend removal for the six rotating conditions with a reported NCC candidate. Gray circles show the original values. Blue squares and brown triangles show the values after removing trends fitted over interval A (150–200 and 300–400 Hz) and interval B (120–200 and 300–450 Hz), respectively. Lines connect values from the same condition. The dashed horizontal line marks the 45° threshold. All values use the full dataset and are evaluated at the same original phase-change peak and window within each condition. This peak may differ from the nearby FRF magnitude peak defining the candidate frequency. Meeting the phase-change threshold alone does not validate a candidate as a structural mode.

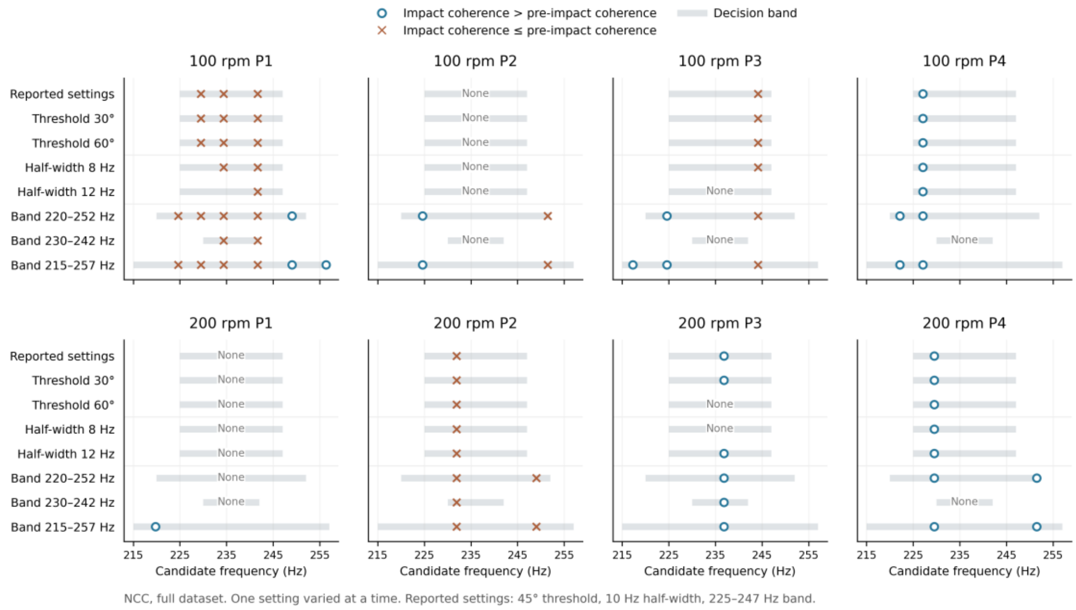

Figure S11. Sensitivity of candidate detection and pre-impact comparison to analysis settings. Each panel represents one rotational speed and excitation position. Rows show the reported settings and changes to one parameter at a time. Gray horizontal segments indicate the decision band. Open circles indicate candidates for which impact-response coherence exceeds pre-impact coherence. Crosses indicate candidates for which it does not. “None” denotes no detected candidate. The reported settings are a 45° phase-change threshold, a 10 Hz phase-window half-width, and a 225–247 Hz decision band. All results use NCC and the full dataset. All detected candidates are shown, rather than only the representative candidate reported for each condition. The figure presents selected comparisons from the 36-combination analysis. Symbols indicate only the pre-impact comparison and do not include the phase-trend removal test. An open circle does not independently validate a structural mode.

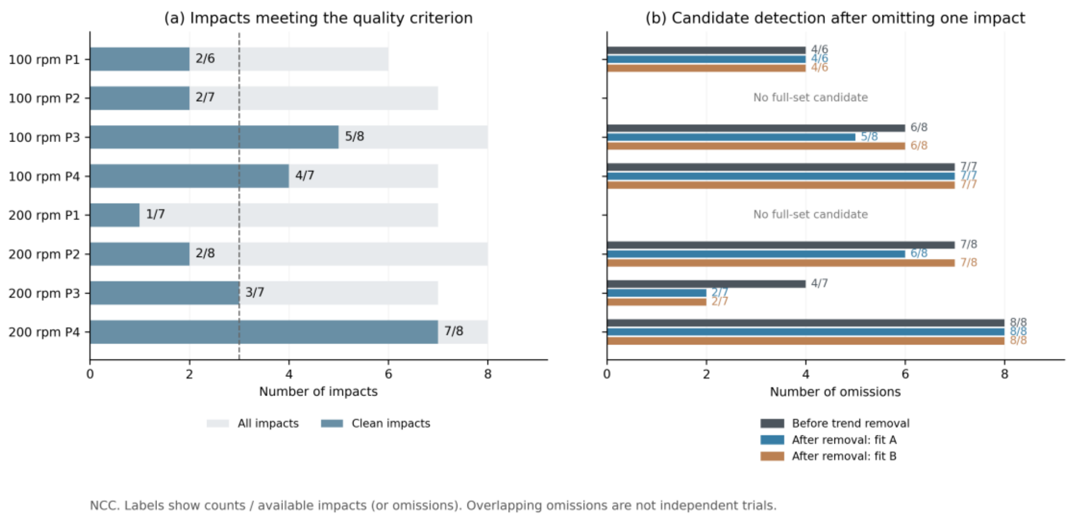

Figure S12. Impact-quality screening and sensitivity to individual impact omissions. (a) Total and clean-impact counts for each condition. The dashed line marks the minimum of three clean impacts required for the reported comparison. (b) Numbers of omissions returning a candidate before and after linear phase-trend removal. Fitting intervals A and B are 150–200 plus 300–400 Hz and 120–200 plus 300–450 Hz, respectively. Fractions use the total number of impacts in panel (a) and the total number of omissions in panel (b) as their denominators. Omissions with no redetected candidate are not counted as retained. Candidate frequencies may differ between omissions. Panel (b) shows candidate detection and phase-trend outcomes, not the pre-impact comparison or independent validation of structural modes. All results use NCC. The overlapping leave-one-out subsets are not independent trials.
